# Testosterone promotion effect of *Eucommia ulmoides* staminate flower via the steroidogenic pathway and potential hormonal mechanism

**DOI:** 10.1038/s41598-022-23578-y

**Published:** 2022-11-05

**Authors:** Zihan Li, Ping Yang, Shan Xue, Shijun Yuan, Lin Yuan, Renyi Yan, Ding Tang, Juan Li

**Affiliations:** 1 Hubei Province Key Laboratory of Traditional Chinese Medicine Resource and Chemistry, Department of Pharmacy, Hubei University of Chinese Medicine, Huang‑Jia‑Hu West Road 16#, Hongshan District, Wuhan, 430065 Hubei China; 2 Hubei Provincial Key Laboratory of Occurrence and Intervention of Rheumatic Diseases, Hubei Minzu University, Enshi, 445000 Hubei China; 3 Central Laboratory, Huanggang Hospital of Traditional Chinese Medicine, Huanggang, Hubei China; 4 Tianjin Ubasio Technology Group Co., Ltd., Tianjin, China

**Keywords:** Endocrine system and metabolic diseases, Cell biology

## Abstract

*Eucommia ulmoides* staminate flowers (EUF), a newly approved functional food in China, have great potential for hormonal regulation. Herein, we aim to demonstrate the chemical composition and pharmacological activity of EUF in testosterone production and hormonal regulation. EUF extract and its components, kaempferol and geniposidic acid, exhibited a strong stimulating effect by increasing testosterone secretion, reducing ROS production, or promoting viability in Leydig cells. Meanwhile, the increased testosterone production was related to the upregulation of mRNA and protein expression of the steroidogenic pathway, such as steroidogenic acute-regulatory protein (StAR), 3β -hydroxysteroid dehydrogenase type 1 (HSD3B1), 17α-hydroxylase/17,20-lyase (CYP17A1), and nuclear receptor subfamily 5 group A member 1 (NR5A1). However, PKA inhibitor H89 or adenylyl cyclase inhibitor SQ22536 could block their effect. The results of transgenic yeast models showed the androgenic agonistic effects of kaempferol and naringenin and the estrogenic agonistic effects of rutin. These results indicated that the testosterone promotional effect of EUF was related to the activation of the steroidogenic pathway and potential hormonal regulation. Kaempferol and geniposidic acid might be the key active ingredients.

## Introduction

*Eucommia ulmoides* Oliver, a well-known and monotypic species of the Eucommia, is widely used as a traditional medicinal plant in Asia with a long history. It has been cultivated in nearly three hundred and fifty thousand hectares in China, accounting for more than 99% of the world's total resources^1^. As a traditional aphrodisiac, its leaves, stem, bark, and staminate flower can be both exploited^2^. Studies have shown that the water extract of *E. ulmoides* can increase the weight of testis and the number of Leydig cells in mice; it can also enhance serum and penile testosterone levels in diabetic rats^3^. In Korea, the leaves of *E. ulmoides* have been used as a folk remedy for the treatment of diabetes^4^. In 2014, the National Health Commission of China added EUF to their novel food list^5^.

Due to the abundance of nutrients and bioactive components*, E. ulmoides* has a potential role in hormonal effects^6^. The male flowers have higher nutritional value and health-promoting impact than the bark and leaves^7^. It has been reported that flavonoids and triterpenes are the main components of EUF^8^, and the phytochemical investigation provided 27 compounds, including 11 triterpenes, 7 flavonoids, 4 fatty acids, 1 coumarin, 1 chromone, and 3 cyclopeptide alkaloids^9^. It exhibited multiple pharmacological activities, including anti-fatigue, anti-hypertensive, anti-hyperlipidemia, and hypnotic effects^10–14^. However, the research regarding its utilization as an aphrodisiac is still unknown.

Testosterone, a hormone produced via steroidogenesis in testicles, plays an essential role in fetal development and the male reproductive system. Leydig cells are the primary cells of testosterone synthesis and secretion. Although Leydig cells make up only 2% to 4% of the testicular cell population, they can produce 95% of the plasma testosterone content^15–17^. Luteinizing hormone (LH) from the pituitary gland regulates testosterone production in testicular Leydig cells via binding to and activating G protein-coupled receptors. This leads to adenylyl cyclase activation, increased intracellular 3′,5′-cyclic adenosine monophosphate (cAMP) formation, and cAMP-dependent phosphorylation of proteins through PKA. The elevation of cAMP/ PKA stimulates signals of downstream steroidogenic proteins, such as StAR, CYP11A1, HSD3B1 and CYP17A1^18,19^. The present research studied the effects of EUF extract and their nutritional ingredients on Leydig cell testosterone production and steroidogenic signaling pathway using a rat Leydig cells model, suggesting EUF to be a candidate drug for hormonal regulation.

## Materials and methods

### Experimental animals

SPF Sprague–Dawley male rats (7 weeks, n = 27) were purchased from Hubei Experimental Animal Research Center and housed under conditions with controlled temperature (22 °C ± 1 °C) and 12 h light/12 h dark cycle for 7 days to adapt to the new environment. The experimental scheme of rat treatment has been approved by Hubei Experimental Animal Research Center. The Laboratory animal permission number was SCXK 2020–0018. All methods were carried out following relevant guidelines and regulations, and this study was carried out in compliance with the Animal Research: Reporting of In Vivo Experiments (ARRIVE) guidelines.

### Samples

EUF was harvested between March and April, and EUF and the main compounds of EUF were obtained from Wuhan Yibei Biological Company (Wuhan, China). The EUF was extracted by refluxing with water, and then the solution was filtered and concentrated by vacuum drying to powder.

UPLC-MS chromatogram was applied for examining components in EUF extract, and 24 compounds were identified. The content of pinoresinol diglucoside, geniposidic acid, and chlorogenic acid is 0.03, 0.63, and 0.22 mg/mL. Fifteen compounds, including six flavonoids (kaempferol, naringenin, quercetin, rutin, kaempferol-3-*O*-rutinoside, astragalin), five iridoids (chlorogenic acid, isochlorogenic acid A, caffeic acid, pinoresinol diglucoside, ferulic acid), and four phenylpropanoids (geniposidic acid, asperulosidic acid, deacetyl asperulosidic acid, aucubin), were selected to detect their effects on testosterone production in Leydig cells^20^.

### Collection and culture of Leydig cells

Purifying rat Leydig cells was carried out according to Klinefelter GR et al*.* with some modifications^21^. First, rats were euthanized by cervical dislocation after anesthetizing, and testicles were placed in 0.5 g/L collagenases and digested at 37 °C for 30 min. The cell supernatant containing Leydig cells was filtered through a 70 µm nylon mesh. Cells were washed and resuspended by DMEM/F12 medium. The cells were added to the prefabricated Percoll concentration gradient centrifuge (5%, 30%, 58%, 70%) at 1650 rpm at 4 °C for 30 min, followed by the collection of the second cell band and added with the medium. The cells were then cultured in DMEM/F12 (Hyclone, USA) supplemented with 8% fetal bovine serum (Gibco, USA), 2% equine serum, and 1% penicillin at 37 °C in a 5% CO_2_ incubator for 24 h before the following experiments.

### Leydig cell purity

According to the reported histochemical method of 3β-hydroxysteroid dehydrogenase (3β-HSD) enzyme staining with some modifications^22,23^. Dyeing reagents were the following: staining solution A (1 mL DMSO with 10 mg DHEA and NBT), staining solution B (1 mL PBS with 10 mg β-NAD), and solution C (1 mL PBS with 1 mg niacinamide). Solutions A, B, C, and PBS were mixed in a ratio of 1:10:10:79. Leydig cells were first digested with trypsin and resuspended with medium (containing serum). After counting with a cell counter, we added 50 μL of the cell suspension and 200 μL of 3β-HSD stain into a 96-well plate, and then the plate was incubated in an incubator for 2 h. The percentage of positively stained cells with distinct blue reaction products was counted under an inverted microscope (Olympus, Tokyo, Japan). Cells with purity greater than 80% after staining with 3β-HSD enzyme were acquired for subsequent experiments.

### CCK-8 assay

The cells were seeded in 96-well culture plates at a density of 10^4^ cells per well, then allowed to attach for 24 h before being treated with varying concentrations of EUF extract (12.5, 25, 50,100 μg/mL) and its monomer ingredients (0, 12.5, 25, 50 μM) for 24 h. Subsequently, 100 µL CCK-8 (Sigma, USA) of 1 mg/mL was added to each well for 2 h. The Optical density of each well at 450 nm was measured with a microplate reader (BioTek Instruments, Inc., USA).

### Testosterone levels detected by ELISA

The cells were seeded in 24-well culture plates at a density of 4 × 10^4^ cells per well and divided into the positive group (10 μM forskolin), sample groups (EUF extract or monomer ingredients for 24 h), and control group. Testosterone concentrations in the medium were measured following the manufacturer's instructions by a rat testosterone ELISA kit (NanjingJiancheng Institute of Biological Engineering, China) (sensitivity: 0.5–150 nmol/L; inter-assay variation: CV < 10%; co-efficient variation: CV < 12%).

### Reactive oxygen species (ROS) measurement

ROS levels were determined by the oxidation of 2′,7′-dichlorofluorescin diacetate into a strong green fluorescent substance (DCF) that could not cross cell membranes. In this experiment, cells were divided into the control group, the Tert-butyl hydroperoxide (TBHP) group (model group), and the TBHP + drug group. ROS was measured following the manufacturer's instructions by a Reactive Oxygen Species Assay Kit (Elabscience, China).

### RT-qPCR analysis

Cells were cultured separately in EUF extract, kaempferol, and geniposidic acid for 24 h. RNA was extracted from the cells following the instructions of the RNeasy Plus Mini RNA extraction kit (Tiangen, China). Synthesis of cDNA was performed on a PCR amplifier following the reverse transcription kit manual. Semi-quantitative real-time PCR was performed in a Fast Real-Time PCR system (LightCycler 480II, Roche, Switzerland). The primer sequences used in this study were as Table 1. The electrophoresis results were observed on a gel imaging system (FCQ, ProteinSimple, USA).

**Table 1:** Sequences of primers used for RT-PCR.

Gene	Forward primer (3′ → 5′)	Reverse primer (5′ → 3′)
*Gapdh*	GGCTCTCTGCTCCTCCCTGT	CGTTCACACCGACCTTCACC
*Star*	GGAACCCAAATGTCAAGGAAATCA	CAGGCATCTCCCCAAAGTGTG
*Cyp11a1*	GTCCAGTTGGTCCCACTCCTC	AAGCACCAGGTCGTTCACAATATAC
*Hsd3b1*	GTACATTTATGGGGAGAGAAGTCC	CCAGGCCACATTGCCTACATA
*Cyp17a1*	GCTCCGAAGGGCAAGTAA	TCCGAGAAGTGCTGCGTAT
*Nr5a1*	TCTCTAACCGCACCATCAAG	TCGACAATGGAGATAAAGGTC

### Western blot analysis

In this experiment, cells were divided into different groups: control group, 10 µM forskolin group, different EUF extract groups (100, 50, and 25 µg/mL), kaempferol or geniposidic acid groups (50, 25, 5 µM) for 24 h. Inhibitor + different drug groups (10 µM H89 or SQ22563 pretreatment for 1 h).

Total protein extracts for western blot analysis were prepared using lysis buffer. Protein concentration was measured by the BCA method. Western blotting was performed as described in our previous study^24^. Primary antibodies against StAR, HSD3B1, NR5A1, CYP11A1, CYP17A1 and GAPDH (ABclonal, Wuhan, China) were used at a 1:1000–1:2000 dilution in 2 ml per band. Subsequently, the bands were incubated with secondary antibodies (1:2000, HRP-conjugated anti-rabbit IgG, GB23303) for 1 h at room temperature. The results were detected with the ECL reagent and a gel imaging system (FCQ, ProteinSimple, USA).

### Yeast estrogen screen (YES) and yeast androgen screen (YAS) assay

The oestrogenic, anti-oestrogenic, androgenic, and anti-androgenic activities were measured with the XenoScreen XL YES/YAS Assay kit (Xenometrix, XenoScreen XL YES/YAS) according to the manufacturer’s protocol^25^. The DNA sequence of human estrogen receptor (hER) or human androgen receptor (hAR) is transfected into growing yeast cells (*Saccharomyces cerevisiae*), and the expression of the reporter gene LAC-Z in yeast cells is regulated by estrogen. 17β-estradiol (E2), 4-hydroxytamoxifen (4-HT), 5α-dihydrotestosterone (DHT), and flutamide (FL) were used as the estrogen agonist, estrogen antagonist, androgen agonist, and androgen antagonist controls, respectively. Antagonistic activities were measured by evaluating the β-galactosidase signal reduction in yeast cells in the presence of 0.2 nM E2 (YES) or 1.0 nM DHT (YAS) in the test medium.

### Statistical analysis

All data were presented as the means ± standard deviation from experimental triplicates. GraphPad Prism 8 software was applied to analyze the independent Dunnett’s test. *P* < 0.05 or *P* < 0.01 was considered a statistically significant difference.

## Results

### Leydig cells purity and morphology

**Figure 1:**
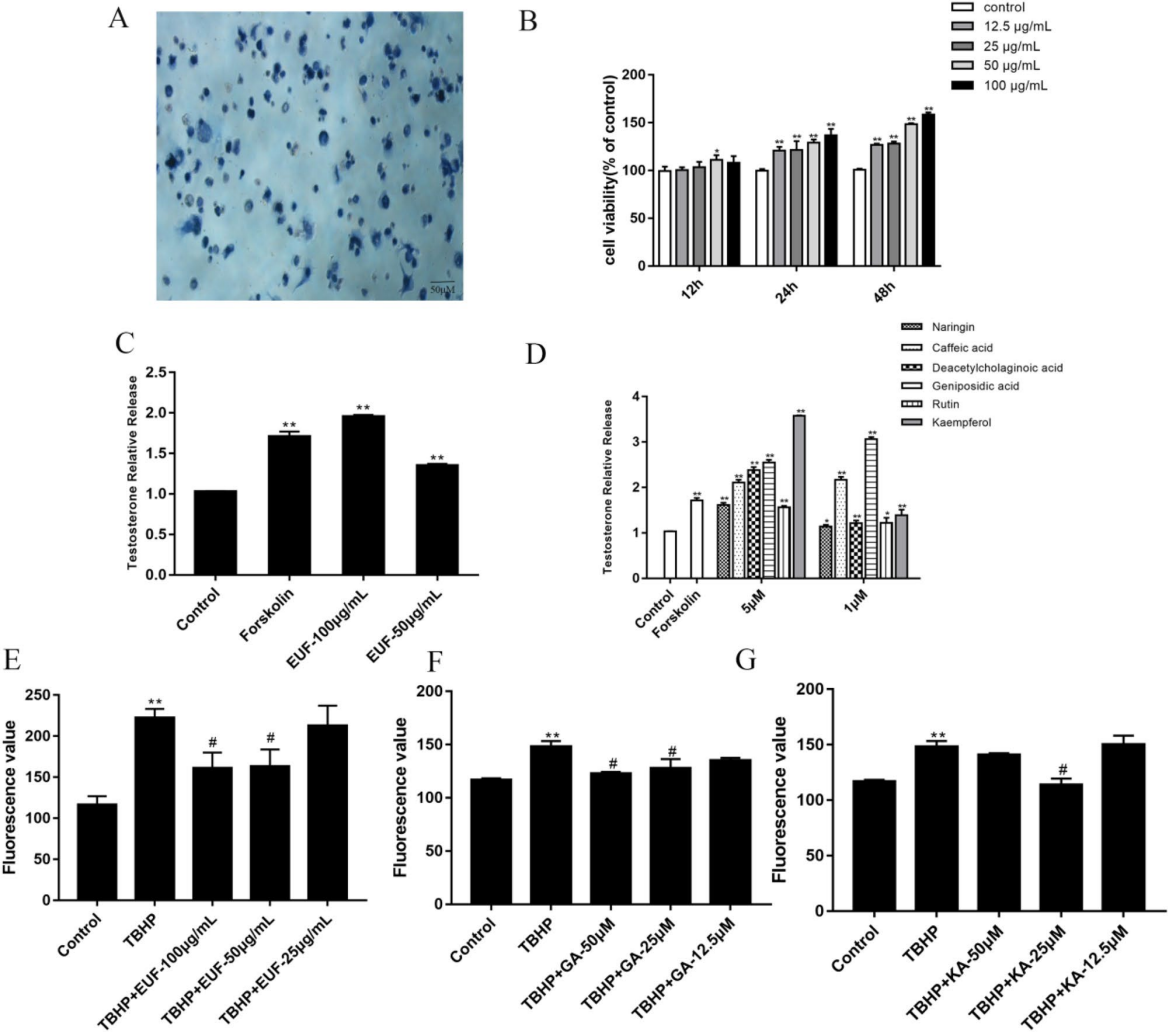
Effect of EUF on testosterone secretion in Leydig cells. (**A**) 3β-HSD staining of Leydig cells (200 ×). (**B**) Effect of EUF extract on the viability of Leydig cells. (**C**) Effect of EUF extract on the testosterone content. (**D**) Effect of monomer on the testosterone content. (**E**–**G**) EUF extract, geniposidic acid and kaempferol decreased TBHP-induced intracellular ROS levels. versus the controls: **P < 0.01, *P < 0.05; versus the TBHP group: ^##^ *p* < 0.01, ^#^*p* < 0.05. KA, kaempferol; GA, geniposidic acid.

The Leydig cells were fusiform, triangular, or polygonal, with a large nucleus and cell cord arrangement, presenting the "pulling net." The cytoplasm of most of the cells was bluish-black with long antennae, while the cytoplasm of a few cells was pale and greyish-blue. The results showed that the calculated purity reached more than 80% by 3β-HSD enzyme dyeing (Fig. 1A).

### Effect of EUF on the cell viability

As shown in Fig. 1B and Table 2, there was no significant difference in the compound groups except for isochlorogenic acid A, geniposidic acid, and EUF extract, compared with the control group. EUF extract, isochlorogenic acid A, and geniposidic acid had a noticeable effect on promoting cell viability in a dose-dependent and time-dependent manner.

**Table 2:** Effects of main compounds in EUF on the survival rate of cell Leydig viability (%).

No.	Compounds	50 μM	25 μM	12.5 μM
1	Kaempferol-3-O-rutinoside	102.24 ± 0.02	98.72 ± 0.01	97.27 ± 0.03
2	Asperuloside acid	99.16 ± 0.02	102.42 ± 0.02	99.21 ± 0.03
3	Chlorogenic acid	101.83 ± 0.1	100.91 ± 0.15	108.92 ± 0.06
4	Isochlorogenic acid A	109.11 ± 0.07	110.59 ± 0.14	127.96 ± 0.04**
5	Aucubin	100.46 ± 0.1	97.55 ± 0.10	101.22 ± 0.10
6	Caffeic acid	102.55 ± 0.03	103.85 ± 0.03	103.54 ± 0.01
7	Deacetyl asperulosidic acid	106.02 ± 0.01	103.74 ± 0.02	98.08 ± 0.11
8	Ferulic acid	110.30 ± 0.13	116.34 ± 0.07	109.30 ± 0.06
9	Astragalin	97.13 ± 0.08	105.86 ± 0.07	109.83 ± 0.07
10	Naringenin	104.68 ± 0.15	105.71 ± 0.03	93.81 ± 0.16
11	Pinoresinol diglucoside	103.07 ± 0.06	105.67 ± 0.03	99.46 ± 0.04
12	Geniposidic acid	142.75 ± 0.06**	115.02 ± 0.06	110.02 ± 0.03
13	Rutin	116.5 ± 0.11	107.13 ± 0.03	110.45 ± 0.03
14	Kaempferol	108.16 ± 0.03	119.48 ± 0.02	113.42 ± 0.02
15	Quercetin	90.05 ± 0.05	98.99 ± 0.02	109.2 ± 0.02
16	Control	100.00 ± 0.08		

*P < 0.05, **P < 0.01 versus the control groups.

### EUF increased testosterone production in Leydig cell

The results showed that forskolin significantly promoted testosterone content (*P* < 0.01). Compared with the control group, EUF extract, caffeic acid, deacetylcholaginoic acid, naringin, geniposidic acid, rutin, and kaempferol could promote testosterone secretion in Leydig cells at 1 μM and 5 μM. Geniposidic acid and kaempferol showed more substantial effects than others (Fig. 1C,D).

### Changes in intracellular ROS levels after EUF extract, kaempferol, and geniposidic acid treatment

As shown in Fig. 1E-G, compared with the control group, ROS levels in the TBHP group were significantly increased, indicating that the oxidative stress model was successfully established (*P* < 0.01). On the other hand, EUF extract, geniposidic acid, and kaempferol groups significantly decreased ROS content compared with the TBHP group (*P* < 0.05). Thus, results indicated that EUF extract, geniposidic acid and kaempferol could attenuate cellular ROS accumulation.

### EUF extract, kaempferol, and geniposidic acid changed mRNA expression of steroidogenic enzyme

**Figure 2:**
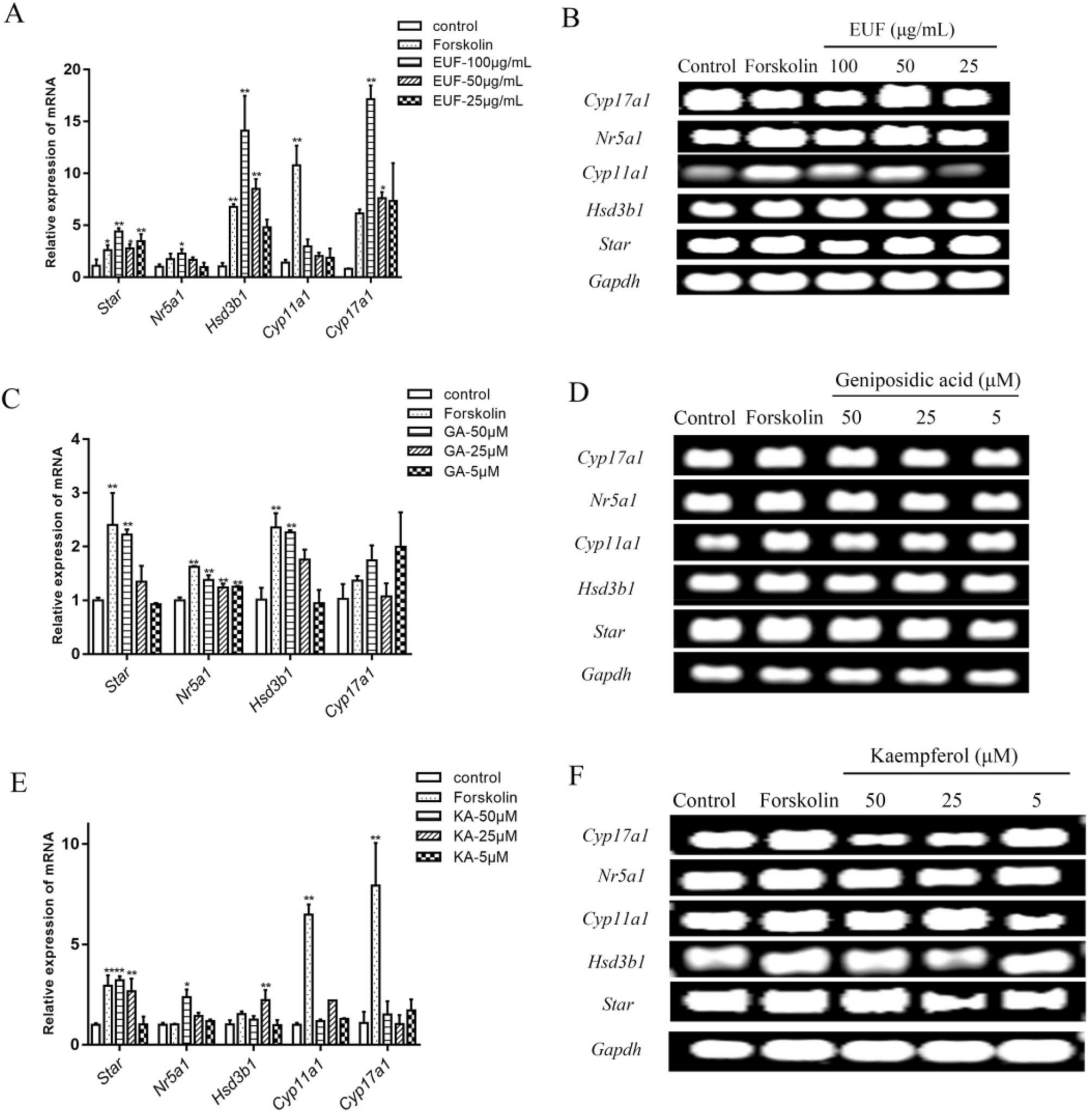
Effects of EUF extract (**A**), geniposidic acid (**C**), and kaempferol (**E**) on the mRNA expression of the steroidogenic enzyme in Leydig cells**.** Agarose gel electrophoresis results of RT-qPCR amplification products (**B**, **D**, **F**). *P < 0.05, **P < 0.01 versus the controls. EUF, *E. ulmoides* staminate flowers; KA, kaempferol; GA, geniposidic acid.

We investigated the mRNA expression levels of steroidogenic pathway genes (*Star*, *Nr5a1*, *Cyp11a1*, *Cyp17a1*, and *Hsd3b1*) in Leydig cells. The fusion curves of these genes were all single peaks without hetero peaks, indicating that the five target genes and reference genes were all specifically expressed. The results of RT-qPCR showed that the 100 µg/mL EUF extract had the best effect on promoting the mRNA expression of *Star*, *Nr5a1*, *Cyp11a1*, *Cyp17a1*, and *Hsd3b1* in Leydig cells (*P* < 0.01). Meanwhile, the expression of the *Cyp11a1* gene was promoted, but there was no significant difference. (Fig. 2A,B).

The results of RT-qPCR showed that *Star*, *Hsd3b1*, and *Nr5a1* mRNA expression levels were significantly increased (*P* < 0.05) in the geniposidic acid group in a dose-dependent manner compared with controls. In addition, 50 µM kaempferol could markedly promote the expression of *StAR* and *Nr5a1* genes. It could also significantly promote the expression of *Hsd3b1* and *Cyp11a1* genes at 25 µM. (Fig. 2C-F).

### EUF extract, kaempferol, and geniposidic acid changed protein expression of steroidogenic enzyme

**Figure 3:**
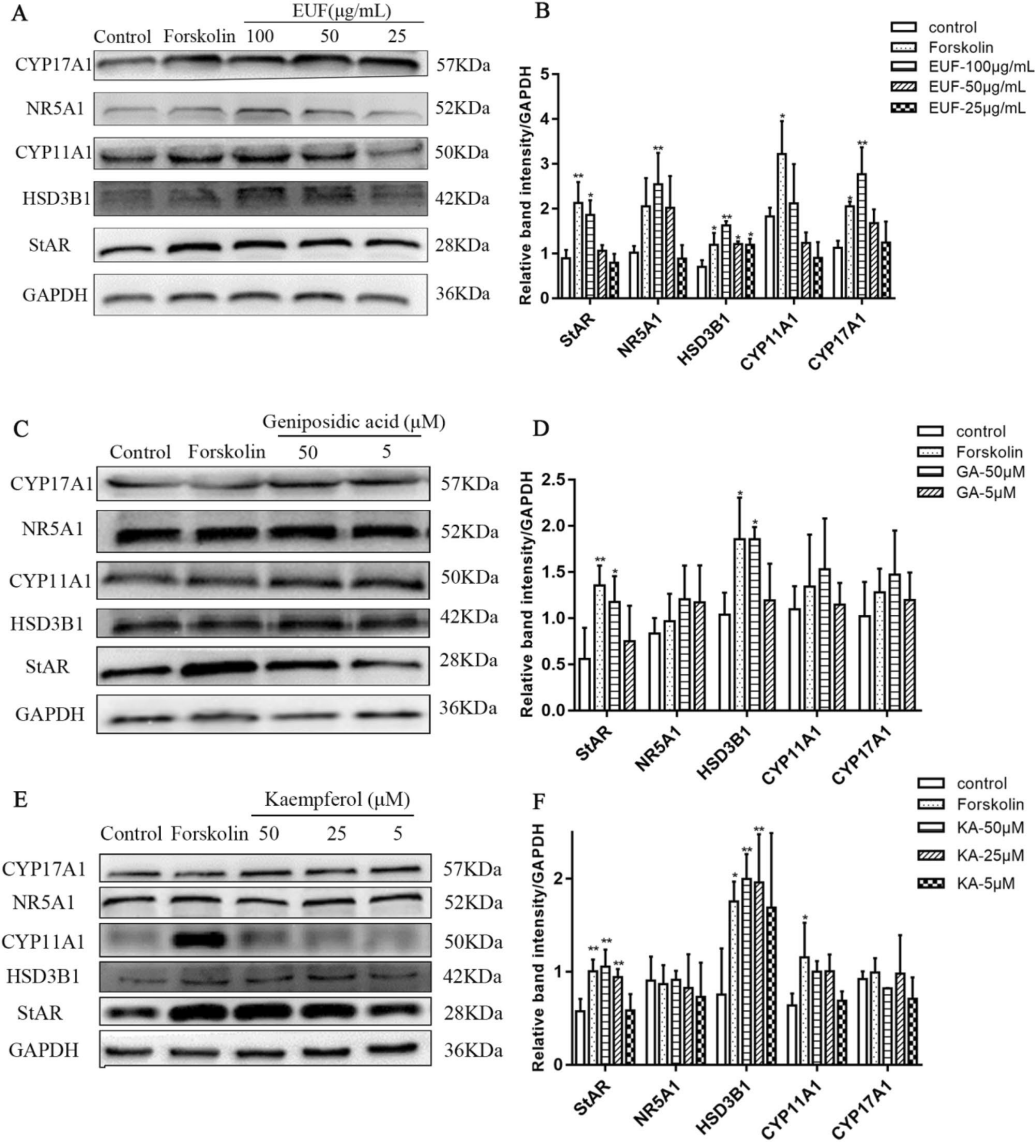
Effects of EUF extract (**A**, **B**), geniposidic acid (**C**, **D**), and kaempferol (**E**, **F**) on the protein expression of the steroidogenic enzyme in Leydig cells. *P < 0.05 versus the controls: **P < 0.01 versus the controls. EUF, *E. ulmoides* staminate flowers.

We performed western blot assays to determine the protein levels of StAR, HSD3B1, CYP17A1, CYP11A1, and NR5A1. Compared with the control group, 100 µg/mL EUF extract could significantly promote the protein expression of StAR, HSD3B1, and CYP17A1 (*P* < 0.01) but had no significant effect on the expression of CYP11A1 (Fig. 3A,B). The protein levels of StAR, HSD3B1, and CYP11A1 were significantly increased in the kaempferol and geniposidic acid group. Meanwhile, geniposidic acid could also promote the protein expression of CYP17A1 and NR5A1 (Fig. 3C-F).

### Effects of EUF on the cAMP-PKA signaling pathway

**Figure 4:**
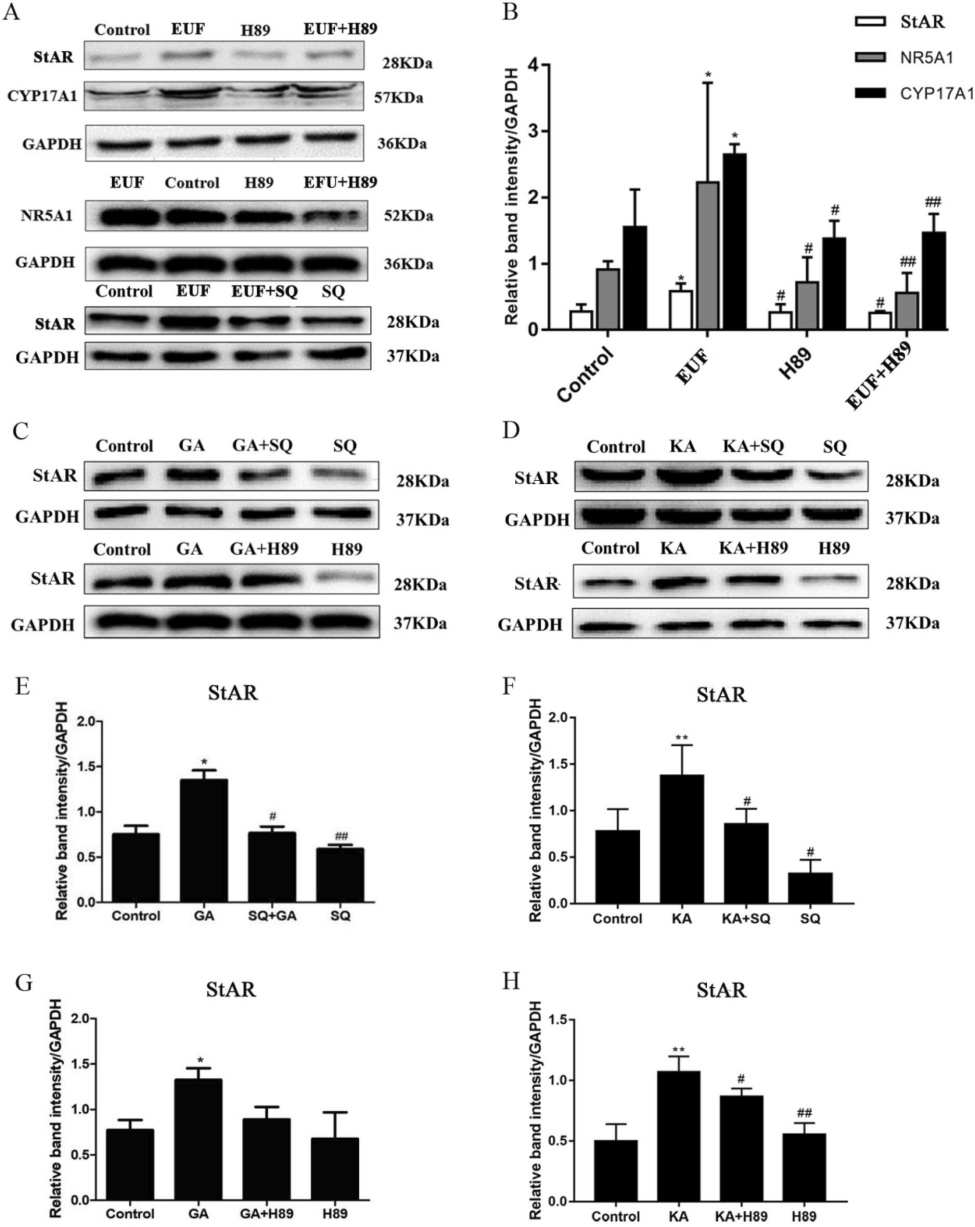
Effects of EUF extract (**A**,**B**), kaempferol, and geniposidic acid (**C**–**H**) on protein expression of the steroidogenic pathway in Leydig cells under the treatment of inhibitors. *P < 0.05, **P < 0.01 versus the control group. ^#^*P* < 0.05, ^##^ *P* < 0.01 versus the EUF, GA, or KA group. KA, kaempferol; GA, geniposidic acid.

As shown in Fig. 4, PKA inhibitor H89 and adenylyl cyclase inhibitor SQ22536 could inhibit the promotion of EUF extract and kaempferol on StAR, and H89 could also inhibit the promotion effect of EUF extract on the expression of CYP17A1 and NR5A1. Geniposidic acid could up-regulate the expression of StAR protein after the SQ22536 treatment but had no significant impact on the expression of StAR protein after the H89 treatment. Thus, the effect of EUF extract, kaempferol and geniposidic acid on promoting androgen synthesis may be closely related to the steroidogenic pathway.

### XenoScreen YES/YAS assay

**Figure 5:**
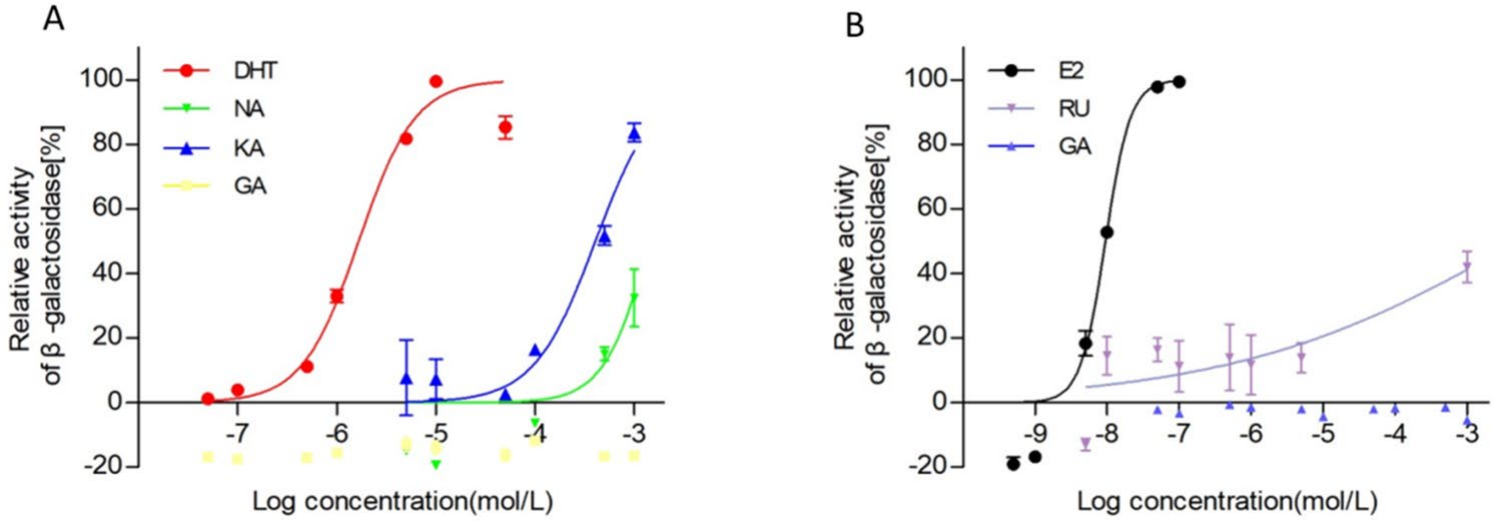
Androgenic (**A**)/estrogenic (**B**) activity of kaempferol, naringenin and rutin. KA, kaempferol; GA, geniposidic acid; NA, naringenin. RU, rutin.

The XenoScreen XL YES/YAS assay^25^ (Table 3, Fig. 5) revealed that, among the fifteen compounds, only kaempferol (7.88 × 10^–4^) and naringenin (8.97 × 10^–4^ ) had androgenic agonistic effects, and rutin had the estrogenic agonistic effect (5.26 × 10^–3^ ). No compounds showed ER or AR antagonistic activities.

**Table 3:** ER and AR agonistic effects (EC50 values).

Group	Sample	EC50(M)
ER agonists	E2	7.89 × 10^–9^
	Rutin	5.26 × 10^–3^
AR agonists	DHT	1.38 × 10^–6^
	Naringenin	8.97 × 10^–4^
	Kaempferol	7.88 × 10^–4^

*ER* estrogenic receptor, *AR* androgenic receptor, *E2* 17β-estradiol, *DHT* 5α-dihydrotestosterone.

## Discussion

Phenylpropanoids, iridoids, and flavonoids are the reported active constituents of *E. ulmoides*^20^. The total flavonoid content in EUF was significantly higher than in other parts (leaves, bark, and fruits) of *E. ulmoides*^26^. The present study showed that EUF extract and its main compounds, including caffeic acid, deacetylcholaginoic acid, naringin, geniposidic acid, rutin, and kaempferol, could stimulate testosterone production. Geniposidic acid and kaempferol showed similar effects with the positive control forskolin. Considering that geniposidic acid and kaempferol have a higher concentration and more significant activities in EUF, they might be the main active components in EUF.

Testosterone synthesis is mainly regulated by the classic LH-LHR-cAMP-PKA pathway of testosterone synthesis. It's regulated by LH height and Leydig cell membranes of G protein-coupled receptor (LH receptor-LHR), activation of adenylyl cyclase, promotes the generation of cAMP, catalytic ATP by PKA phosphorylation. Then, testosterone is synthesized under the action of a series of processes such as StAR, CYP11A1, CYP17A1, and HSD3B1. As an inhibitor of adenylyl cyclase, SQ22536 down-regulates the synthesis of cAMP and then inhibits the activities of testosterone biosynthesis-related proteins such as StAR and NR5A1^27^. H89 could inactivate PKA target protein sites and down-regulate the phosphorylation of downstream proteins such as StAR^28^. The results showed that EUF extract, geniposidic acid, and kaempferol could significantly promote the expression of testosterone synthesis genes or proteins. After pretreatment with SQ22536 and H89, 100 μg/mL EUF extract, 50 μM kaempferol, and 50 μM geniposidic acid could significantly increase testosterone synthetase StAR expression. Therefore, EUF extract, kaempferol, and geniposidic acid raised testosterone synthetase protein expression by activating steroidogenic signaling pathways.

Studies have shown that kaempferol, a phytoestrogen possessing estrogen receptor stress inhibitory activity, could inhibit carcinogenesis and cancer progression^29^. Besides, it could stimulate estrogen signaling followed by the WNT/β-catenin pathway to induce osteoblasts differentiation^30^, activate the opening of BK ca channels in human umbilical vein endothelial cells and strengthen insulin secretory capacity of beta cells through the cAMP/PKA pathway^31,32^. These were similar to our results of the androgenic agonistic effects of kaempferol and naringenin in the XenoScreen XL YES/YAS assay. It has been reported that geniposidic acid showed a protective and promotional effect on primary cultured endothelial cells in an atherosclerotic model^33^. *Eucommiae* Cortex fractions had potent induction of growth hormone release. The cortex fractions and its active component, geniposidic acid, can participate in activating osteoblasts to facilitate osteogenesis and suppressing osteoclast activity to inhibit osteolysis^34^. Similar to the report, we found that geniposidic acid and EUF extract significantly promoted the viability of the Leydig cell.

Here, it could be concluded that EUF extract, kaempferol, and geniposidic acid could stimulate testosterone levels, evidenced by reducing ROS production and promoting Leydig cell viability or raising testosterone secretion. Besides, the active ingredients showed potential hormonal activity on transgenic yeast and upregulation of the steroidogenic pathway. The effect was blocked by PKA inhibitor H89 or adenylyl cyclase inhibitor SQ22536 in Leydig cells, suggesting that their stimulatory mechanism was associated with activating the steroidogenic pathway. It is the first time to elucidate the effect, pharmacodynamic material basis, and mechanism of EUF in testicular testosterone production.

## Supplementary Information

Supplementary Information 1.

Supplementary Information 2.

## Data Availability

The datasets used and analyzed during the current study are available from the corresponding author upon reasonable request.

## Abbreviations

AR: Androgenic receptor
CYP11A1: Cytochrome P450 cholesterol side-chain cleavage
CYP17A1: 17α-Hydroxylase/17,20 lyase
cAMP: 3′,5′-Cyclic adenosine monophosphate
DHT: 5α-Dihydrotestosterone
E2: 17β-Estradiol
ER: Estrogenic receptor
EUF: *Eucommia ulmoides* staminate flowers
FL: Flutamide
GA: Geniposidic acid
hAR: Human androgen receptor
hER: Human estrogen receptor
HSD3B1: 3β-Hydroxysteroid dehydrogenase type 1
4-HT: 4-Hydroxytamoxifen
KA: Kaempferol
LH: Luteinizing hormone
NA: Naringenin
NR5A1: Nuclear receptor subfamily 5 group A member 1
PKA: Protein kinase A
RT-PCR: Real-time reverse transcription polymerase chain reaction
ROS: Reactive oxygen species
StAR: Steroidogenic acute-regulatory protein
TBHP: Tert-butyl hydroperoxide
YAS: Yeast androgen screen
YES: Yeast estrogen screen
