# Supplementary material for: Testosterone promotion effect of Eucommia ulmoides staminate flower via the steroidogenic pathway and potential hormonal mechanism
Source: Sci Rep. 2022 Nov 5;12:18765. doi: 10.1038/s41598-022-23578-y (PMC9637168; doi:10.1038/s41598-022-23578-y)
Supplement: Supplementary file 1 — Supplementary Information 1. [file 41598_2022_23578_MOESM1_ESM.docx]

the Xeno ScreenYES/YAS assay results

|  | Estrogen receptor agonist relative enzyme activity (%) | | | | | |
| --- | --- | --- | --- | --- | --- | --- |
| Log c (M) | E2 | | RU | | GA | |
| -9.3 | -21.344 | -16.906 |  |  |  |  |
| -9 | -18.589 | -15.07 |  |  |  |  |
| -8.3 | 14.47 | 22.215 | -14.8634 | -11.1136 |  |  |
| -8 | 51.955 | 53.558 | 20.40528 | 8.513289 |  |  |
| -7.3 | 97.508 | 98.025 | 19.90181 | 12.69951 | -1.16029 | -3.13573 |
| -7 | 98.935 | 100 | 19.21009 | 3.288302 | -2.4839 | -4.33821 |
| -6.3 |  |  | 24.26714 | 3.690198 | -0.56949 | -0.44524 |
| -6 |  |  | 20.86711 | 2.406298 | -1.58846 | -1.22739 |
| -5.3 |  |  | 9.210767 | 18.54105 | -1.15157 | -3.00766 |
| -5 |  |  |  |  | -4.8437 | -3.81608 |
| -4.3 |  |  |  |  | -1.13169 | -2.75455 |
| -4 |  |  |  |  | -1.27594 | -2.15833 |
| -3.3 |  |  |  |  | -1.56172 | -1.19273 |
| -3 |  |  | 37.11935 | 46.7591 | -6.53944 | -4.36851 |

|  | Androgen receptor agonist relative enzyme activity (%) | | | | | | | |
| --- | --- | --- | --- | --- | --- | --- | --- | --- |
| Log c (M) | DHT | | NA | | KA | | GA | |
| -7.3 | 2.26 | 0.021 |  |  |  |  | -17.748 | -15.8668 |
| -7 | 4.161 | 3.517 |  |  |  |  | -18.0237 | -16.9087 |
| -6.3 | 9.584 | 12.605 |  |  |  |  | -15.8282 | -18.1087 |
| -6 | 30.965 | 34.981 |  |  |  |  | -15.9104 | -15.4012 |
| -5.3 | 81.635 | 82.023 | -13.783 | -16.136 | 19.4 | -4.031 | -15.134 | -10.6475 |
| -5 | 99.012 | 100 | -20.898 | -18.208 | 13.424 | 1.092 | -11.7688 | -15.9033 |
| -4.3 | 88.872 | 81.79 | -17.195 | -15.774 | 2.604 | 2.39 | -18.1932 | -14.4401 |
| -4 |  |  | -6.619 | -6.242 | 16.686 | 16.265 | -12.3846 | -11.2605 |
| -3.3 |  |  | 12.856 | 17.234 | 48.731 | 54.66 | -15.4197 | -17.732 |
| -3 |  |  | 23.479 | 41.29 | 80.761 | 86.59 | -16.2627 | -16.785 |
