# Supplementary material for: Testosterone promotion effect of Eucommia ulmoides staminate flower via the steroidogenic pathway and potential hormonal mechanism
Source: Sci Rep. 2022 Nov 5;12:18765. doi: 10.1038/s41598-022-23578-y (PMC9637168; doi:10.1038/s41598-022-23578-y)
Supplement: Supplementary file 2 — Supplementary Information 2. [file 41598_2022_23578_MOESM2_ESM.docx]

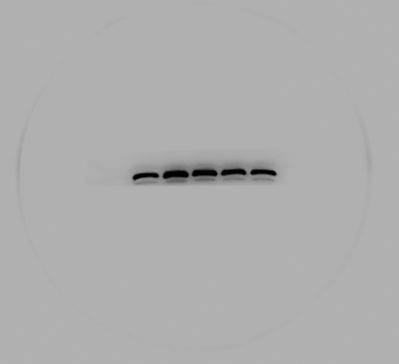

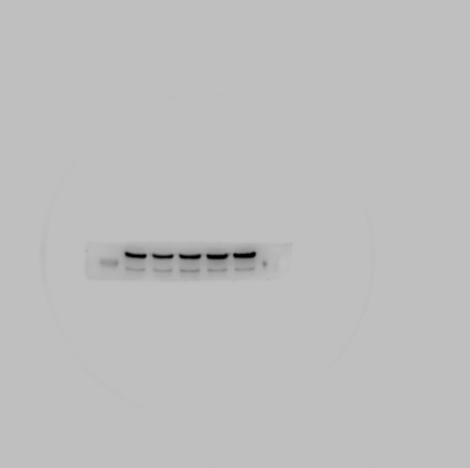
StAR GAPDH

Control, forskolin, 100, 50, 25

Control, forskolin, 100, 50, 25


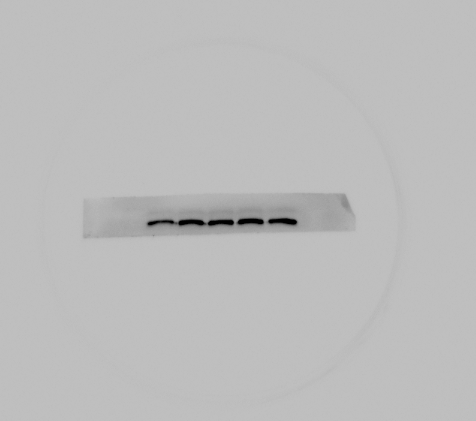

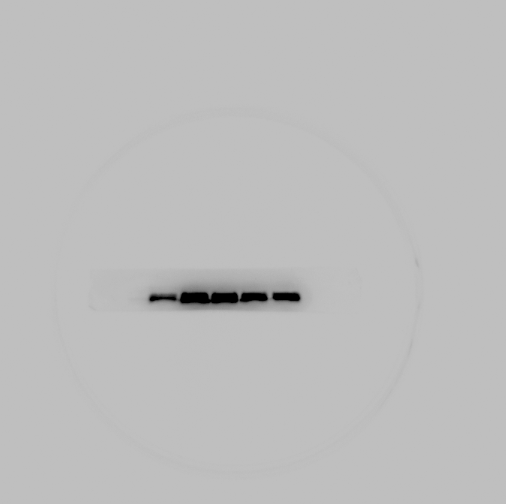
StAR GAPDH

Control, forskolin, 100, 50, 25

Control, forskolin, 100, 50, 25


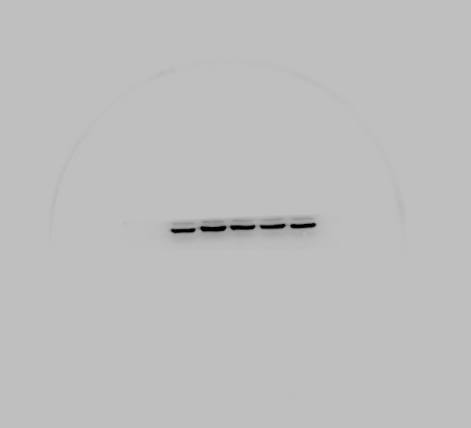

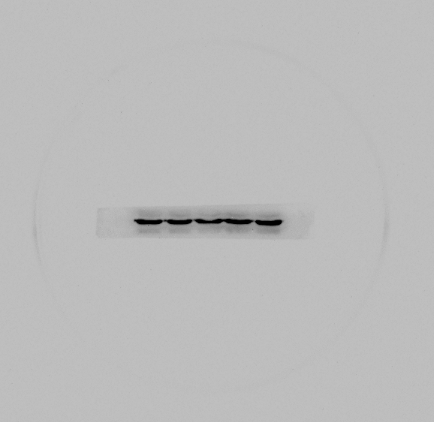
StAR GAPDH

Control, forskolin, 100, 50, 25

Control, forskolin, 100, 50, 25

**Figure. 3A**. Effects of EUF on the protein expression of StAR in Leydig cells. (EUF is E. ulmoides staminate flowers)


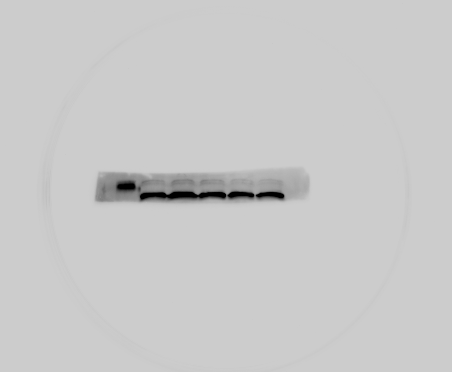

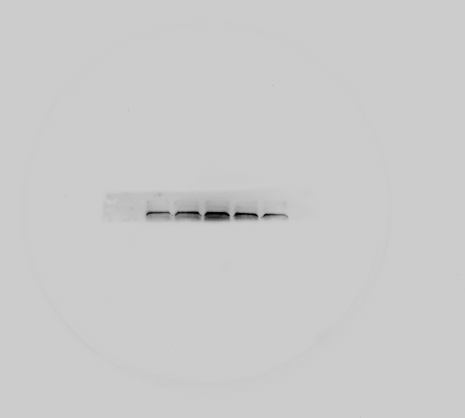
NR5A1 GAPDH

Control, forskolin, 100, 50, 25

Control, forskolin, 100, 50, 25

**
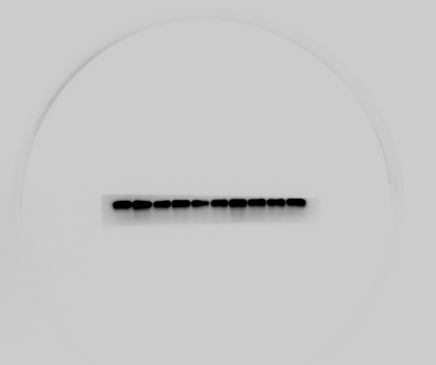

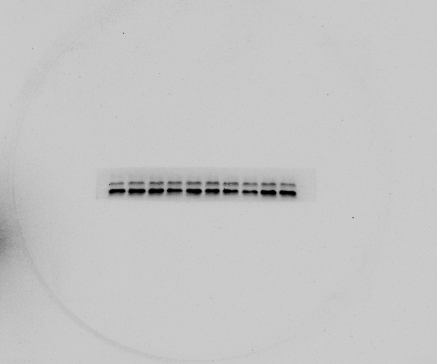

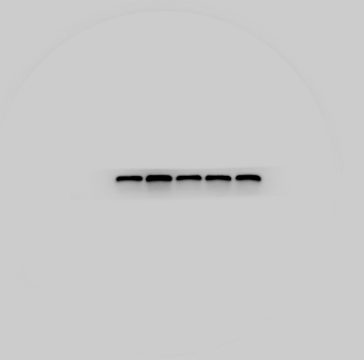
**
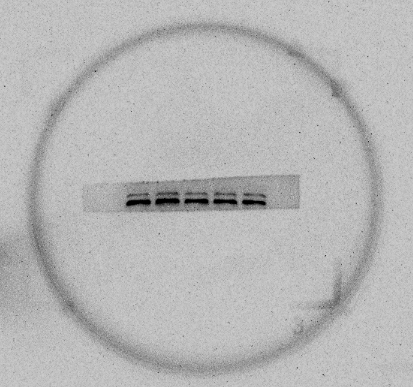
**Figure. 3A**. Effects of EUF on the protein expression of NR5A1 in Leydig cells. (EUF is E. ulmoides staminate flowers)

Control, forskolin, 100, 50, 25,*,*,*,*,*

Control, forskolin, 100, 50, 25

Control, forskolin, 100, 50, 25,*,*,*,*,*

Control, forskolin, 100, 50, 25


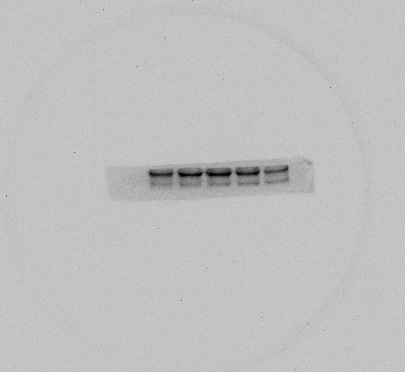

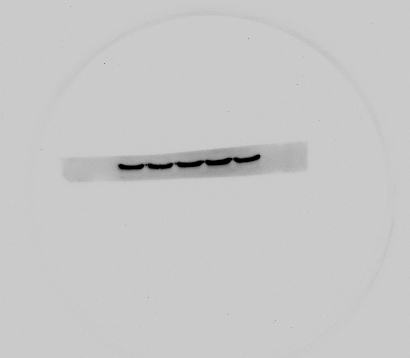
CYP11A1 GAPDH

Control, forskolin, 100, 50, 25

Control, forskolin, 100, 50, 25


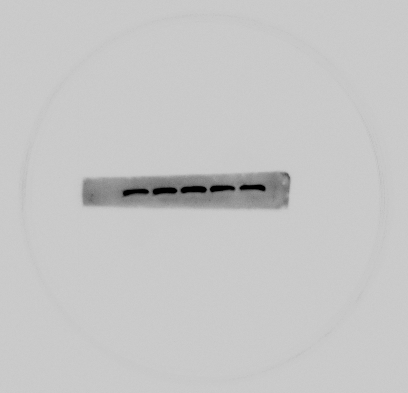

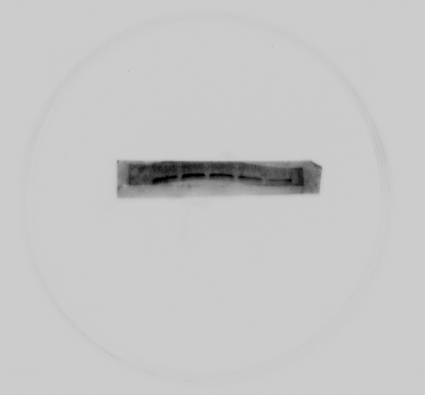
CYP11A1 GAPDH

Control, forskolin, 100, 50, 25

**Figure. 3A**. Effects of EUF on the protein expression of CYP11A1 in Leydig cells. (EUF is E. ulmoides staminate flowers)

Control, forskolin, 100, 50, 25


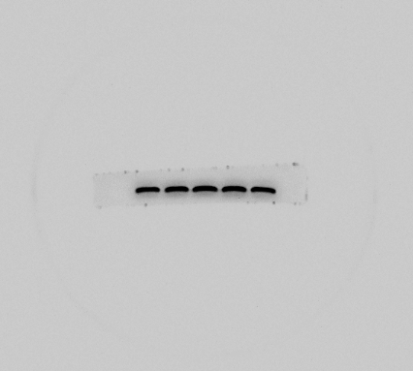

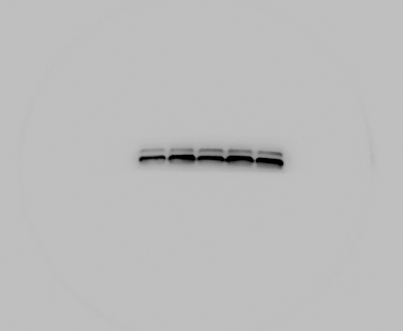
CYP17A1 GAPDH

Control, forskolin, 100, 50, 25

Control, forskolin, 100, 50, 25


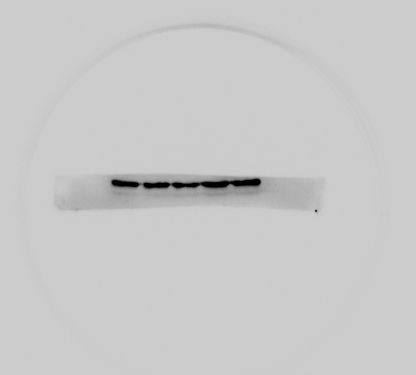

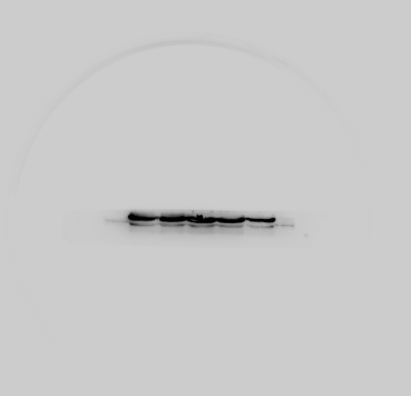
CYP17A1 GAPDH

Control, forskolin, 100, 50, 25

**
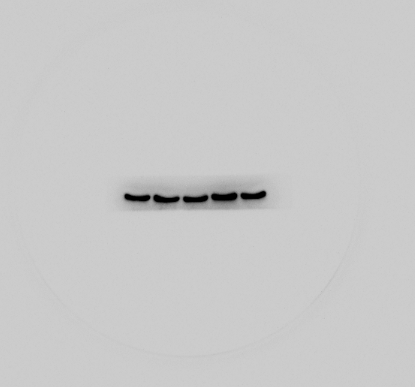

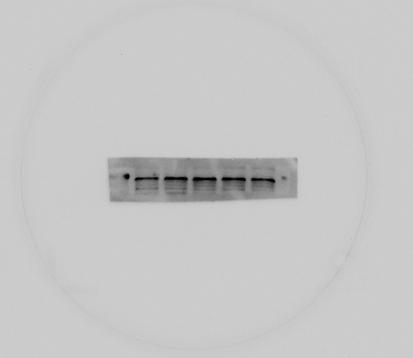
Figure. 3A**. Effects of EUF on the protein expression of CYP17A1 in Leydig cells. (EUF is E. ulmoides staminate flowers)

Control, forskolin, 100, 50, 25

Control, forskolin, 100, 50, 25

Control, forskolin, 100, 50, 25


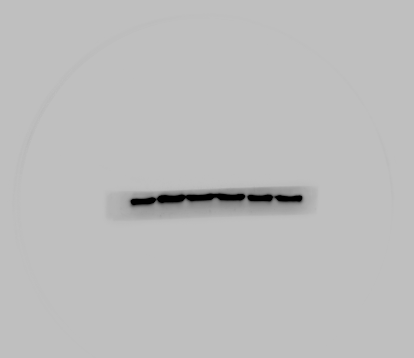

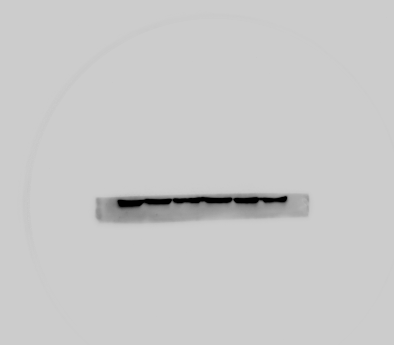
StAR GAPDH


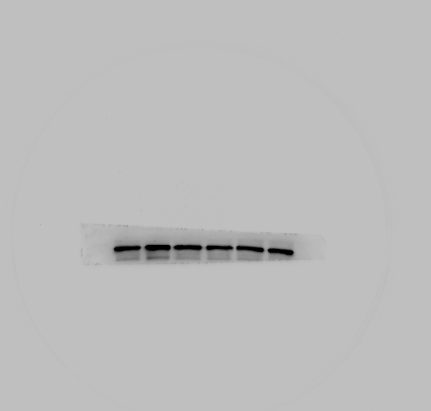


Control, forskolin, 50, 25, *,*

Control, forskolin, 50, 25, *,*


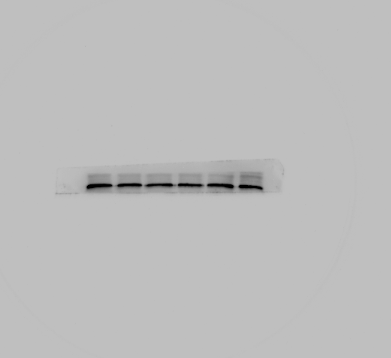
StAR GAPDH

Control, forskolin, 50, 25, *,*

Control, forskolin, 50, 25, *,*


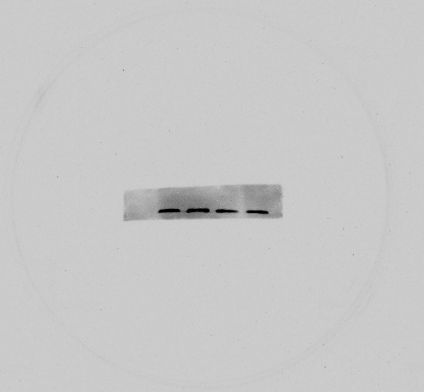

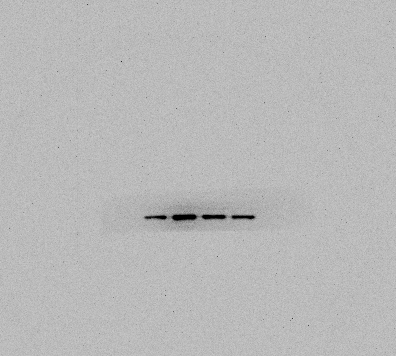
StAR GAPDH

Control, forskolin, 50, 25

Control, forskolin, 50, 25

**Figure. 3B**. Effects of geniposidic acid on the protein expression of StAR in Leydig cells.


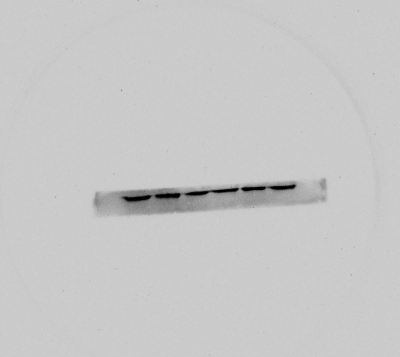

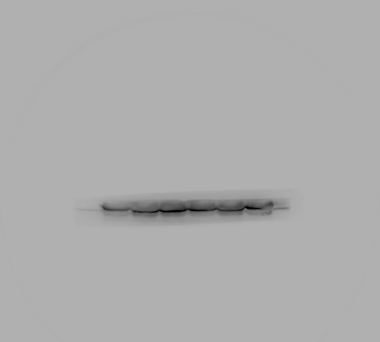
CYP17A1 GAPDH

Control, forskolin, 50, 25, *,*

Control, forskolin, 50, 25, *,*


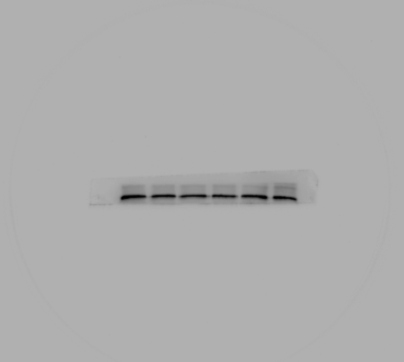

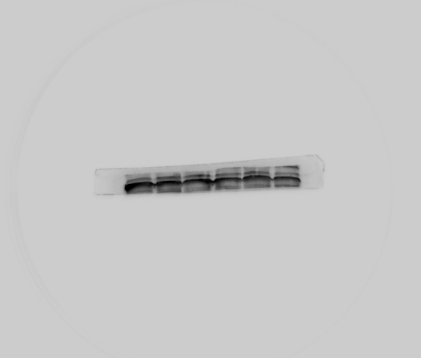
CYP17A1 GAPDH

Control, forskolin, 50, 25, *,*

Control, forskolin, 50, 25, *,*


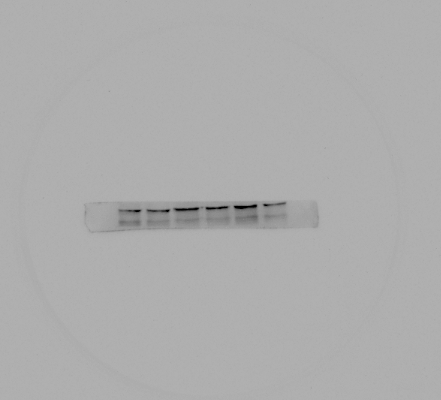

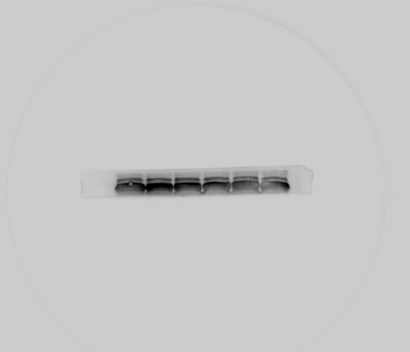
CYP17A1 GAPDH

Control, forskolin, 50, 25, *,*

**Figure. 3B**. Effects of geniposidic acid on the protein expression of CYP17A1 in Leydig cells.

Control, forskolin, 50, 25, *,*


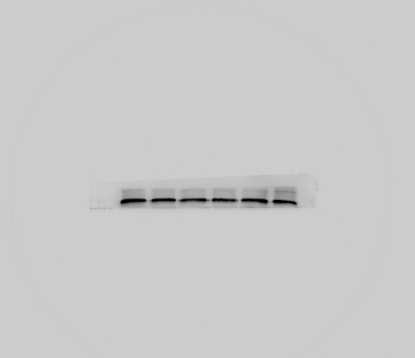

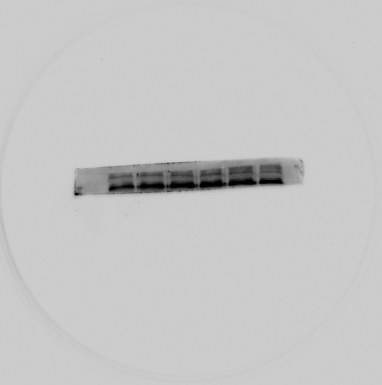
HSD3B1 GAPDH


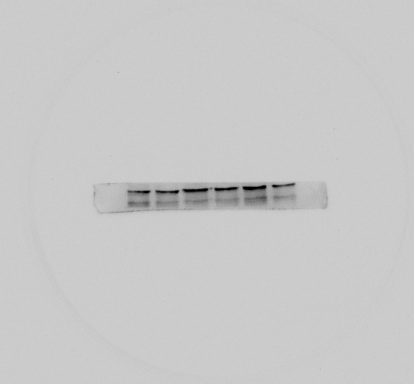


Control, forskolin, 50, 25, *,*

Control, forskolin, 50, 25, *,*


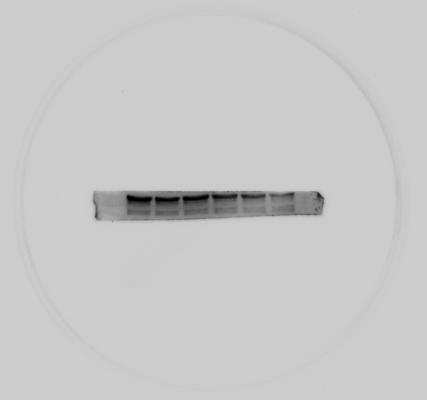
HSD3B1 GAPDH

Control, forskolin, 50, 25, *,*


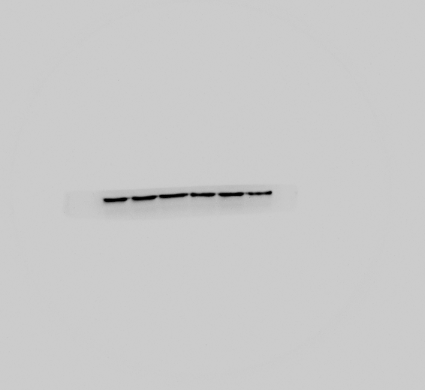

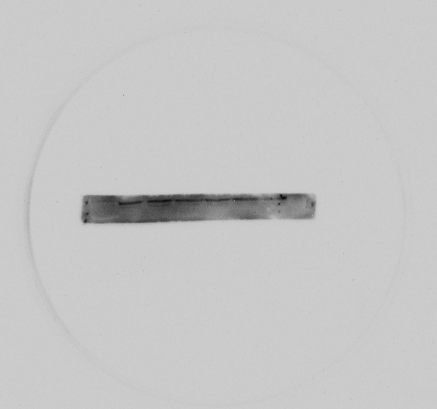
HSD3B1 GAPDH

Control, forskolin, 50, 25, *,*

Control, forskolin, 50, 25, *,*

**Figure. 3B**. Effects of geniposidic acid on the protein expression of HSD3B1 in Leydig cells.

Control, forskolin, 50, 25, *,*


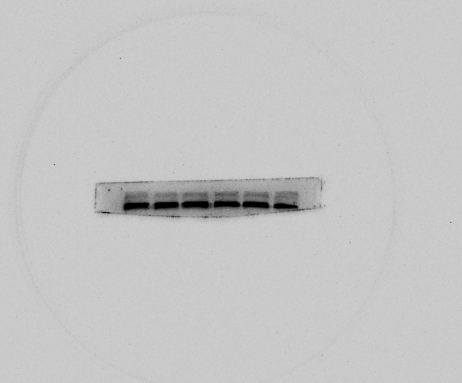

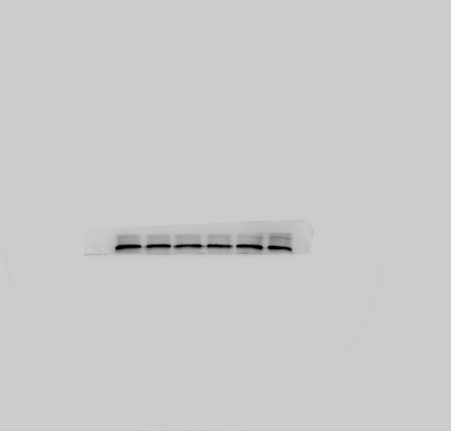
NR5A1 GAPDH

Control, forskolin, 50, 25, *,*


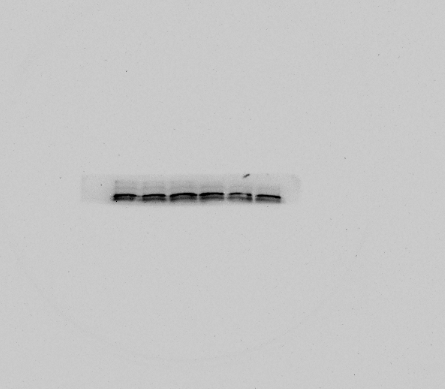

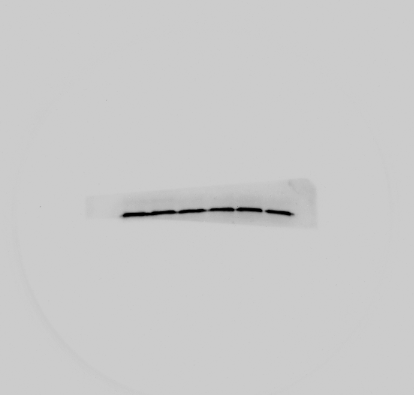


Control, forskolin, 50, 25, *,*

Control, forskolin, 50, 25, *,*

Control, forskolin, 50, 25, *,*


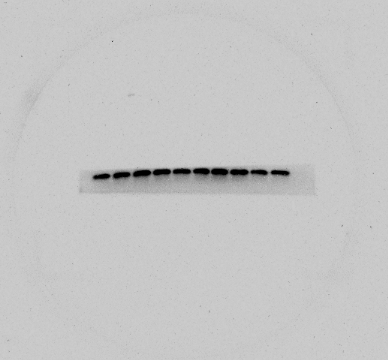

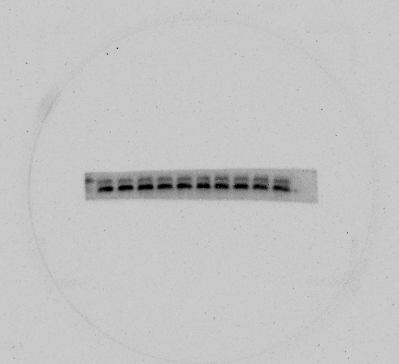


Control, forskolin, 50, 25, *,*

Control, forskolin, 50, 25, *,*,*,*,*,*

**Figure. 3B**. Effects of geniposidic acid on the protein expression of NR5A1 in Leydig cells.


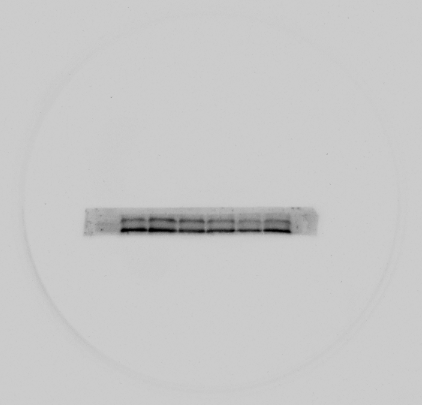

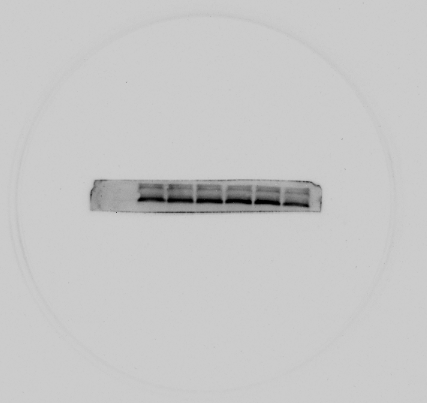

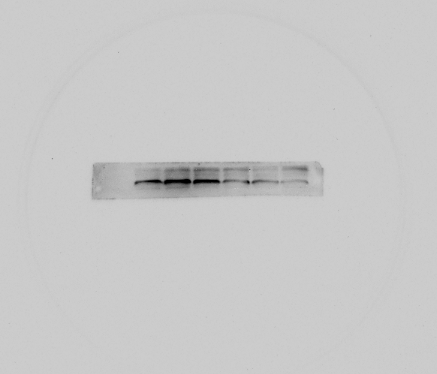
CYP11A1 GAPDH

Control, forskolin, 50, 25, *,*

Control, forskolin, 50, 25, *,*


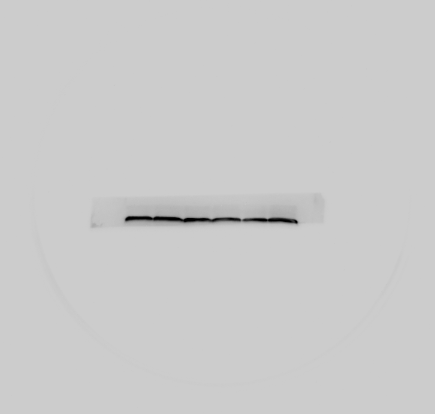
CYP11A1 GAPDH

Control, forskolin, 50, 25, *,*

Control, forskolin, 50, 25, *,*

**Figure. 3B**. Effects of geniposidic acid on the protein expression of CYP11A1 in Leydig cells.


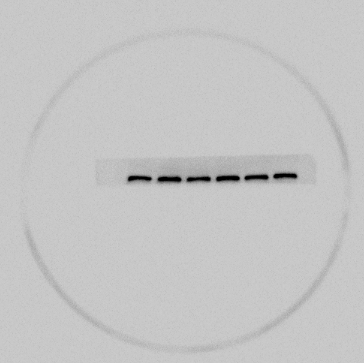

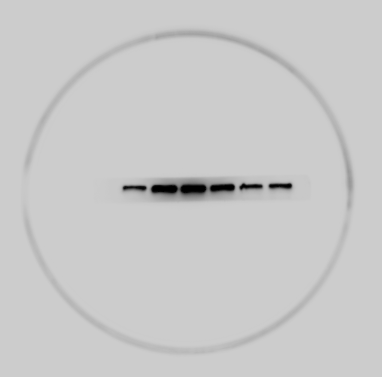
StAR GAPDH


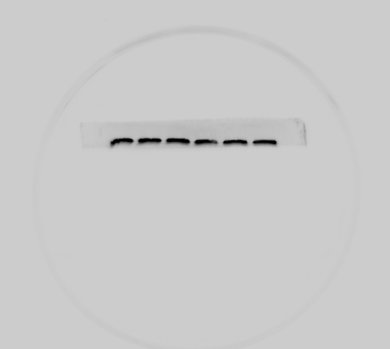

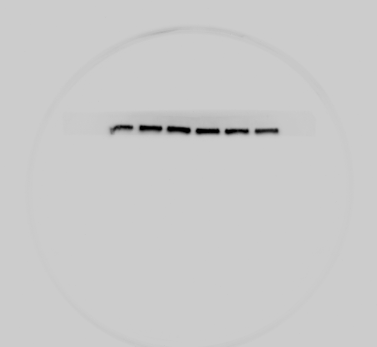
StAR GAPDH

Control, forskolin, 50, 25, 5,*

Control, forskolin, 50, 25, 5,*

Control, forskolin, 50, 25, 5,*


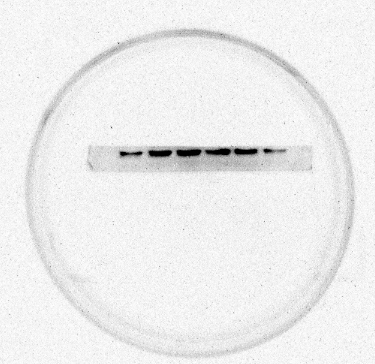

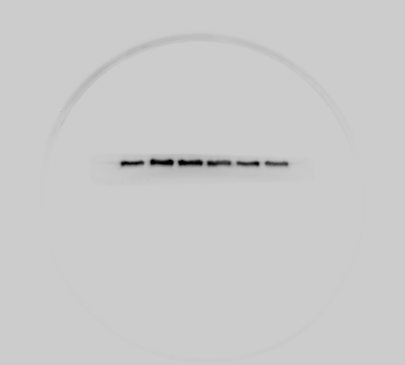
StAR GAPDH

Control, forskolin, 50, 25, 5,*

Control, forskolin, 50, 25, 5,*

**Figure. 3C**. Effects of kaempferol on the protein expression of StAR in Leydig cells.

Control, forskolin, 50, 25, 5,*


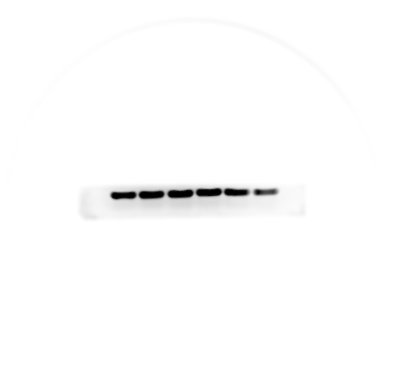

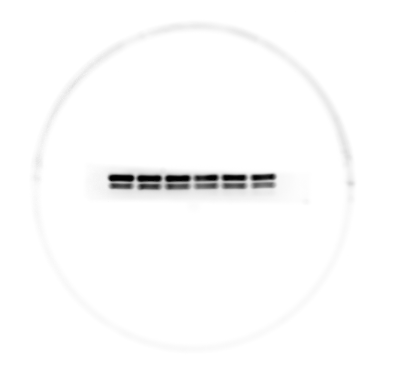
CYP17A1 GAPDH

**
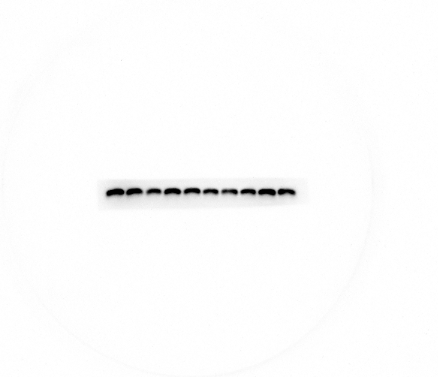

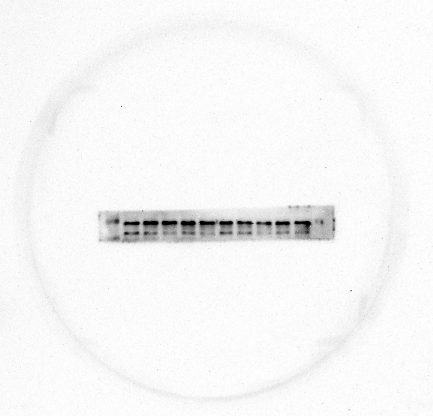
**
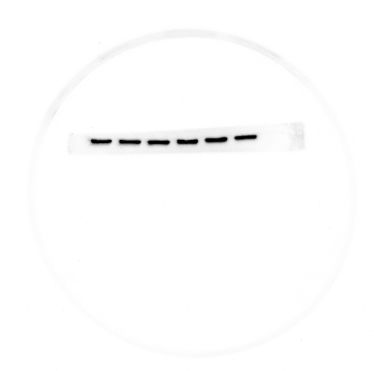

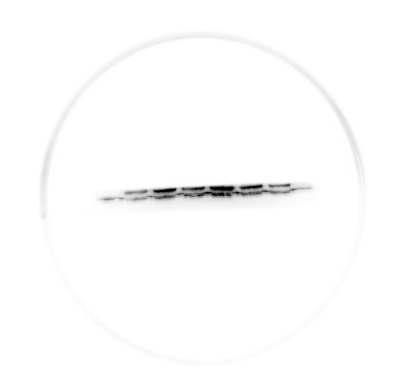
CYP17A1 GAPDH

Control, forskolin, 50, 25, 5,*

Control, forskolin, 50, 25, 5,*

Control, forskolin, 50, 25, 5,*

**Figure. 3C**. Effects of kaempferol on the protein expression of CYP17A1 in Leydig cells.

Control, forskolin, 50, 25, 5,*,*,*,*,*,

Control, forskolin, 50, 25, 5,*,*,*,*,*,

Control, forskolin, 50, 25, 5,*


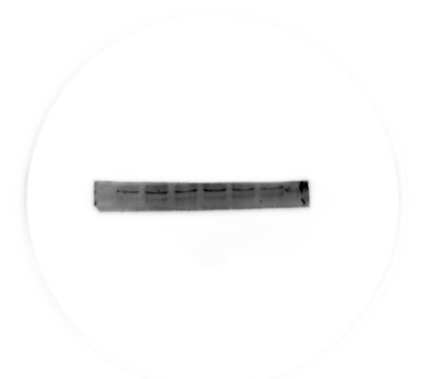

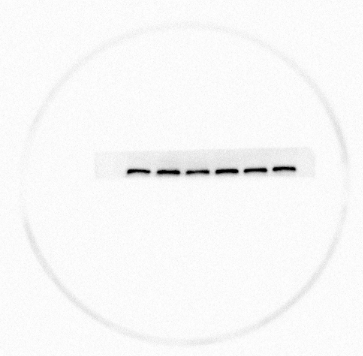
HSD3B1 GAPDH


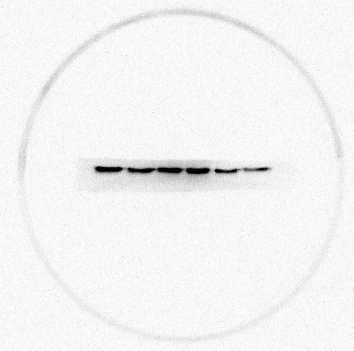

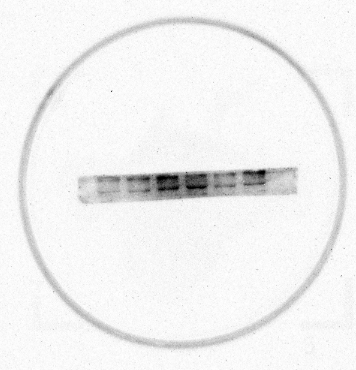
HSD3B1 GAPDH

Control, forskolin, 50, 25, 5,*

Control, forskolin, 50, 25, 5,*

Control, forskolin, 50, 25, 5,*

**Figure. 3C**. Effects of kaempferol on the protein expression of HSD3B1 in Leydig cells.

Control, forskolin, 50, 25, 5,*


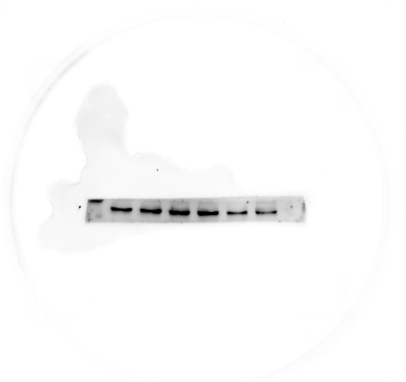

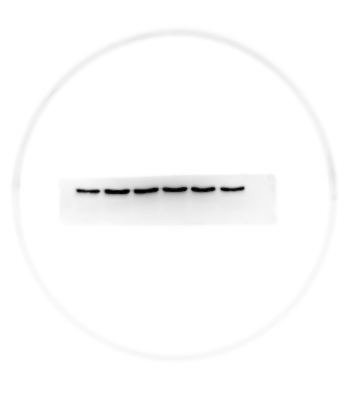
NR5A1 GAPDH


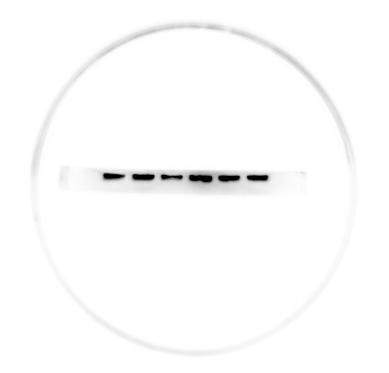


Control, forskolin, 50, 25, 5,*

Control, forskolin, 50, 25, 5,*


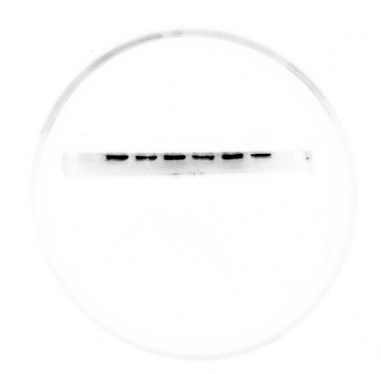
NR5A1 GAPDH

Control, forskolin, 50, 25, 5,*

Control, forskolin, 50, 25, 5,*


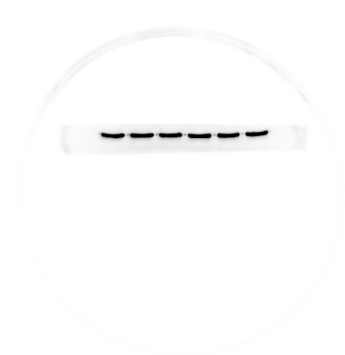

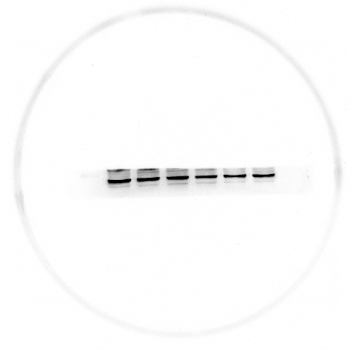
NR5A1 GAPDH

Control, forskolin, 50, 25, 5,*

**Figure. 3C**. Effects of kaempferol on the protein expression of NR5A1 in Leydig cells.

Control, forskolin, 50, 25, 5,*

CYP11A1 GAPDH


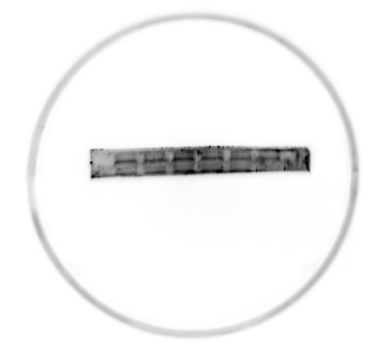

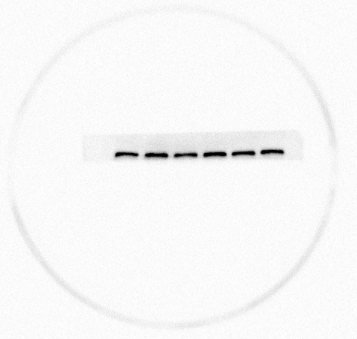


**Figure. 3C**. Effects of kaempferol on the protein expression of CYP11A1 in Leydig cells.

Control, forskolin, 50, 25, 5,*

Control, forskolin, 50, 25, 5,*


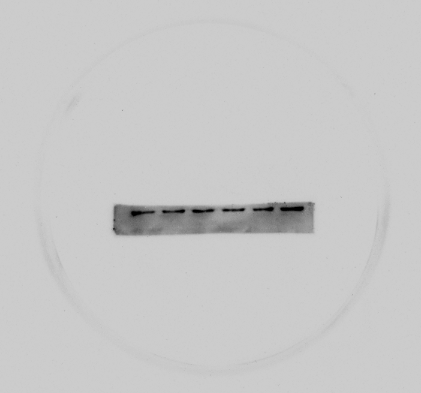

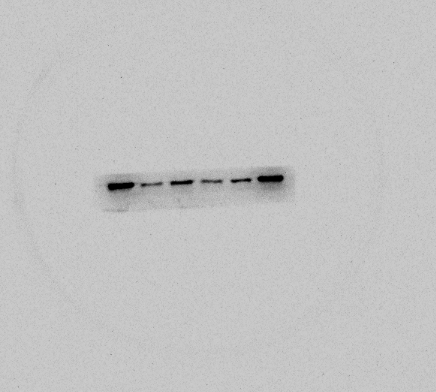
StAR GAPDH

*, Control, EUF, H89, EUF+H89, *


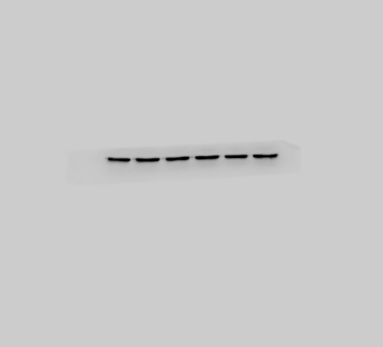


*, Control, EUF, H89, EUF+H89, *


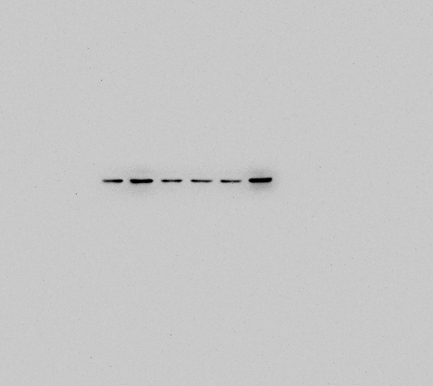
StAR GAPDH


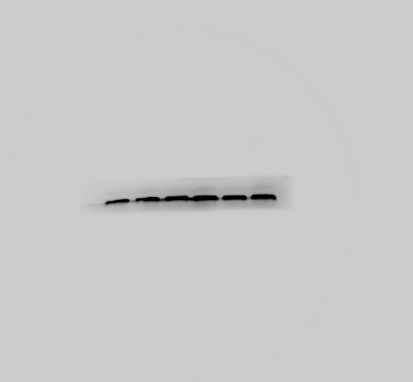

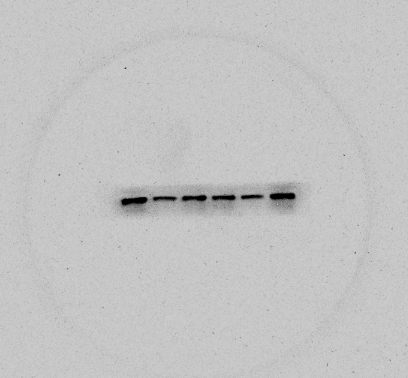
StAR GAPDH

*, EUF, Control, H89, EUF+H89, *

*, Control, EUF, H89, EUF+H89, *

*, EUF, Control, H89, EUF+H89, *

**Figure. 4A**. Effects of EUF on the protein expression of StAR in Leydig cells under the treatment of the inhibitor H89

*, Control, EUF, H89, EUF+H89, *


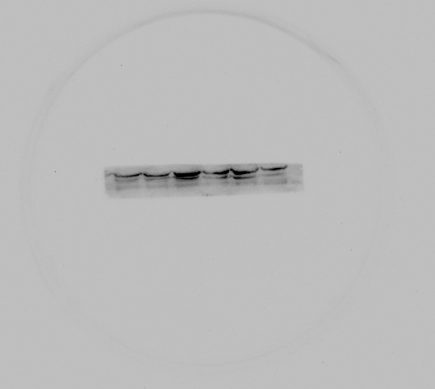

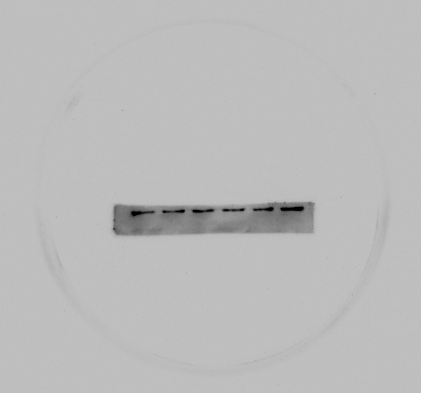
CYP17A1 GAPDH


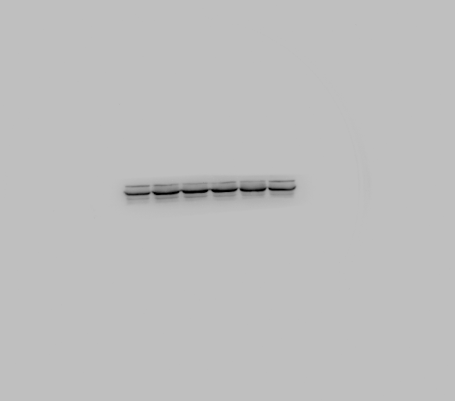

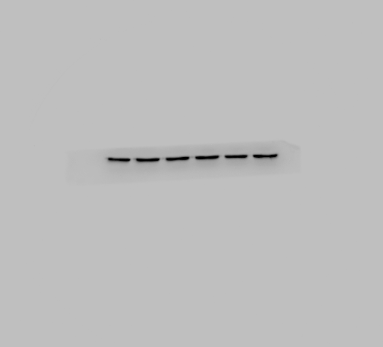
CYP17A1 GAPDH

*, Control, EUF, H89, EUF+H89, *

*, Control, EUF, H89, EUF+H89, *


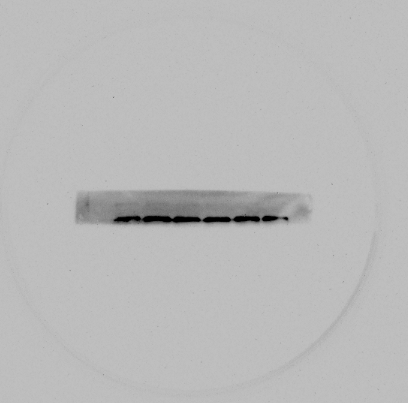

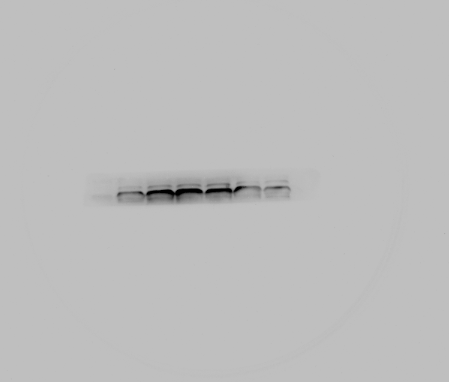
CYP17A1 GAPDH

*, EUF, Control, H89, EUF+H89, *

*, EUF, Control, H89, EUF+H89, *

*, Control, EUF, H89, EUF+H89, *

*, Control, EUF, H89, EUF+H89, *

**Figure. 4A**. Effects of EUF on the protein expression of CYP17A1 in Leydig cells under the treatment of the inhibitor H89


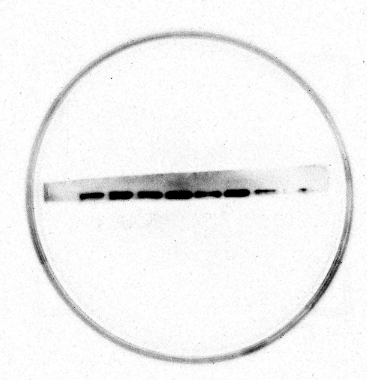

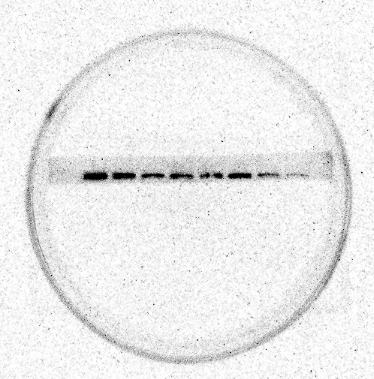
NR5A1 GAPDH


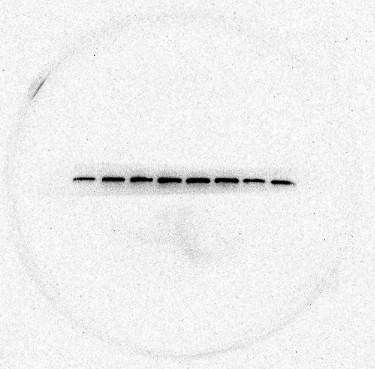

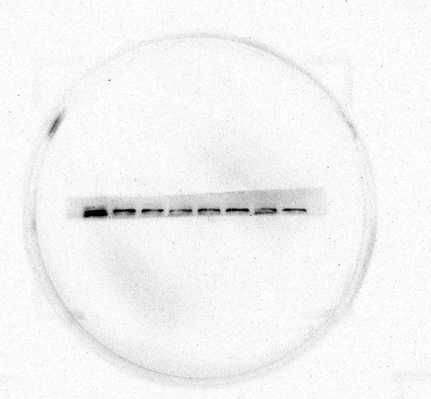
NR5A1 GAPDH

EUF, Control, H89,EUF+H89, *, * ,*,*

EUF, Control, H89,EUF+H89, *, * ,*,*

EUF, Control, H89,EUF+H89, *, * ,*,*

EUF, Control, H89,EUF+H89, *, * ,*,*


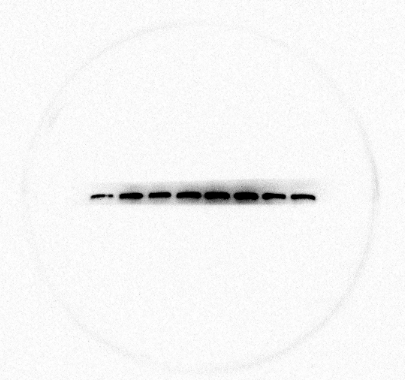

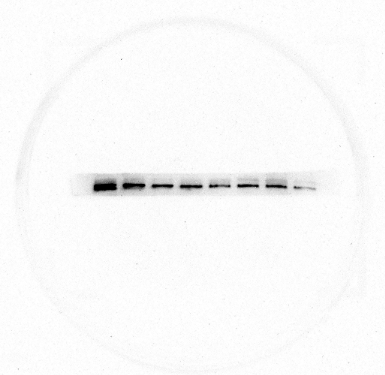
NR5A1 GAPDH

EUF, Control, H89,EUF+H89, *, * ,*,*

**Figure. 4A**. Effects of EUF on the protein expression of NR5A1 in Leydig cells under the treatment of the inhibitor H89

EUF, Control, H89,EUF+H89, *, * ,*,*


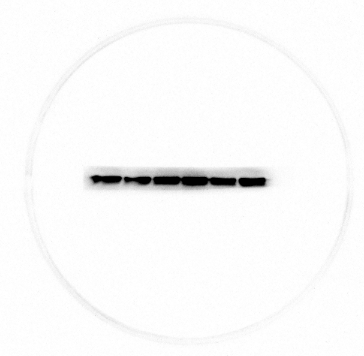
StAR GAPDH


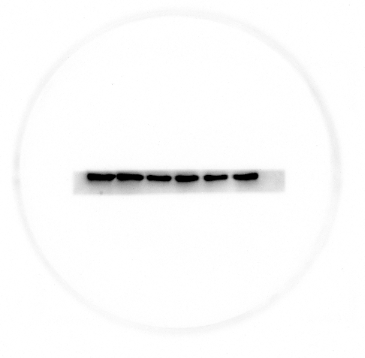

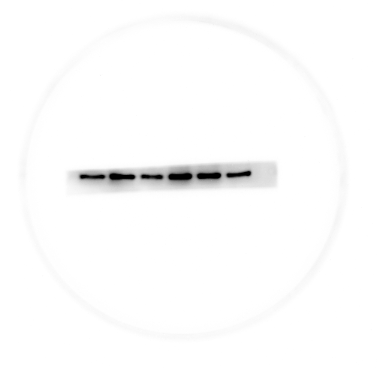
StAR
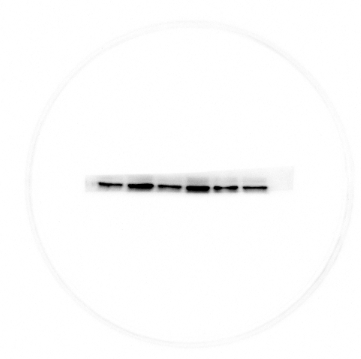
 GAPDH

*,*, Control, EUF, EUF+SQ, SQ,

SQ, EUF+SQ, Control, EUF, *,*,

*,*, Control, EUF, EUF+SQ, SQ,


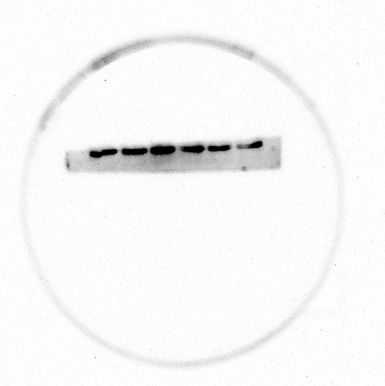

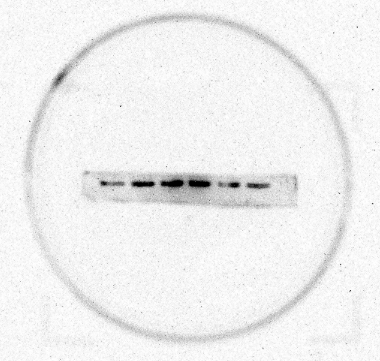
StAR GAPDH

SQ, Control, EUF+SQ, EUF, *,*,

SQ, EUF+SQ, Control, EUF, *,*,

SQ, Control, EUF+SQ, EUF, *,*,

**Figure. 4A**. Effects of EUF on the protein expression of StAR in Leydig cells under the treatment of the inhibitor SQ


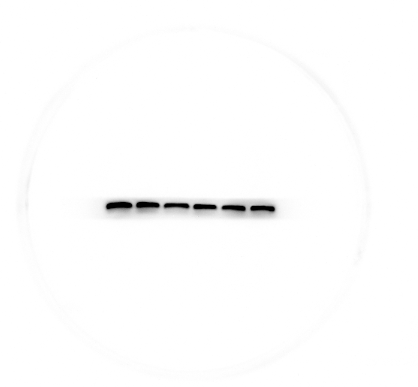

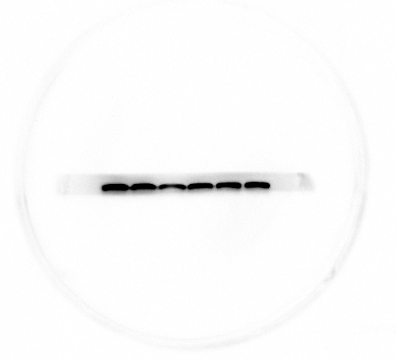
StAR GAPDH

H89, GA+H89, GA, Control *,*,

StAR GAPDH

H89, GA+H89, GA, Control *,*,

H89, GA+H89, GA, Control *,*,

StAR GAPDH

H89, GA+H89, GA, Control *,*,

*,*, GA, Control , GA+H89, H89,

**Figure. 4B**. Effects of geniposidic acid on the protein expression of StAR in Leydig cells under the treatment of the inhibitor H89

*,*, GA, Control , GA+H89, H89,

StAR GAPDH

StAR GAPDH

*,*, Control, GA, GA+SQ, SQ,

*,*, Control, GA, GA+SQ, SQ,

StAR GAPDH

*,*, Control, GA, GA+SQ, SQ,

SQ, GA+SQ, Control, GA, *,*,

*,*, Control, GA, GA+SQ, SQ,

**Figure. 4B**. Effects of geniposidic acid on the protein expression of StAR in Leydig cells under the treatment of the inhibitor SQ

SQ, GA+SQ, Control, GA, *,*,

StAR GAPDH

*,*, Control, KA, KA+ H89, H89,

*,*, Control, KA, KA+ H89, H89,

StAR GAPDH

StAR GAPDH

*,*, Control, KA, KA+ H89, H89,

*,*, Control, KA, KA+ H89, H89,

**Figure. 4B**. Effects of kaempferol on the protein expression of StAR in Leydig cells under the treatment of the inhibitor H89

*,*, Control, KA, KA+H89, H89,

*,*, Control, KA, KA+H89, H89,

StAR GAPDH

*,*, Control, KA, KA+SQ, SQ,

*,*, Control, KA, KA+SQ, SQ,

StAR GAPDH

*,*, Control, KA, KA+SQ, SQ,

*,*, Control, KA, KA+SQ, SQ,

StAR GAPDH

*,*, KA, Control, KA+SQ, SQ,

**Figure. 4B**. Effects of kaempferol on the protein expression of StAR in Leydig cells under the treatment of the inhibitor SQ

*,*, KA, Control, KA+SQ, SQ,
